# *ortho* and *para* chromophores of green fluorescent protein: controlling electron emission and internal conversion†
†Electronic supplementary information (ESI) available: Details of chromophore synthesis and NMR data; potential energy scans along bridge and CCOH torsion; geometrical data of optimised structures; ionisation potentials with state characters; excited state energies and characters; coordinates of optimised geometries. See DOI: 10.1039/c6sc03833f


**DOI:** 10.1039/c6sc03833f

**Published:** 2016-11-07

**Authors:** Conor McLaughlin, Mariana Assmann, Michael A. Parkes, Joanne L. Woodhouse, Ross Lewin, Helen C. Hailes, Graham A. Worth, Helen H. Fielding

**Affiliations:** a Department of Chemistry , University College London , 20 Gordon Street , London WC1H 0AJ , UK . Email: h.h.fielding@ucl.ac.uk

## Abstract

Green fluorescent protein (GFP) continues to play an important role in the biological and biochemical sciences as an efficient fluorescent probe and is also known to undergo light-induced redox transformations. Here, we employ photoelectron spectroscopy and quantum chemistry calculations to investigate how the phenoxide moiety controls the competition between electron emission and internal conversion in the isolated GFP chromophore anion, following photoexcitation with ultraviolet light in the range 400–230 nm. We find that moving the phenoxide group from the *para* position to the *ortho* position enhances internal conversion back to the ground electronic state but that adding an additional OH group to the *para* chromophore, at the *ortho* position, impedes internal conversion. Guided by quantum chemistry calculations, we interpret these observations in terms of torsions around the C–C–C bridge being enhanced by electrostatic repulsions or impeded by the formation of a hydrogen-bonded seven-membered ring. We also find that moving the phenoxide group from the *para* position to the *ortho* position reduces the energy required for detachment processes, whereas adding an additional OH group to the *para* chromophore at the *ortho* position increases the energy required for detachment processes. These results have potential applications in tuning light-induced redox processes of this biologically and technologically important fluorescent protein.

## Abstract 2

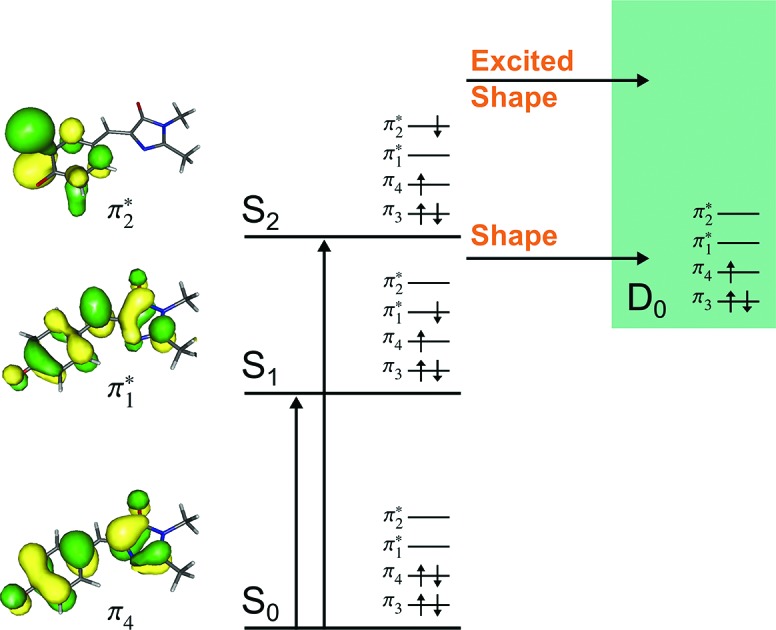
Green fluorescent protein (GFP) plays an important role in the biological and biochemical sciences as an efficient fluorescent probe and as a light-induced electron donor.

## 1 Introduction

**Fig. 1:**
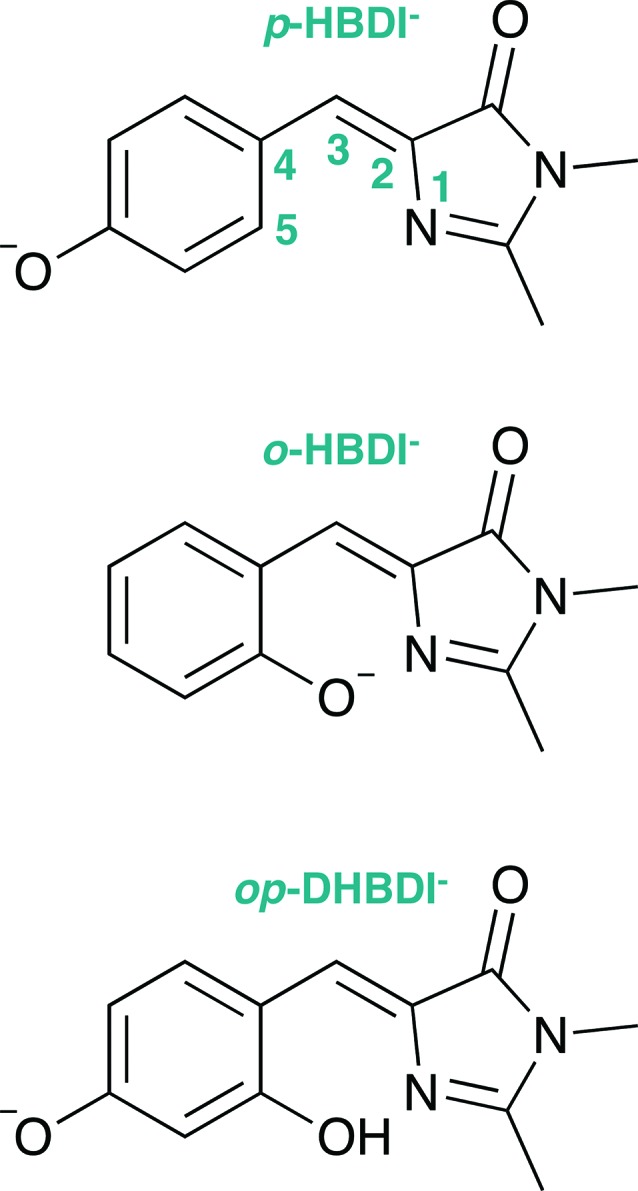
Structures of the model GFP chromophores employed in this work.

Green fluorescent protein (GFP), together with its family of variants, is the most widely used fluorescent protein for *in vivo* monitoring of biological and biochemical processes.^1^–^4^ The chromophore that lies at the heart of GFP is 4-hydroxybenzylidene-1,2-dimethylimidazolinone, *p*-HBDI (the relevant anionic deprotonated form is shown in Fig. 1). The chromophore is anchored, both covalently and *via* a hydrogen-bonded network, to the protein that is wrapped around it in a β-barrel structure. GFP has two absorption bands centered around 395 nm and 480 nm, attributed to the neutral and anionic forms, respectively. Following excitation of the neutral form of the chromophore, excited-state proton transfer takes place *via* a proton relay between water molecules and amino acid residues, resulting in very intense fluorescence (quantum yield *Φ* ≈ 0.8) from the deprotonated anionic form of the chromophore.^5^ This fluorescence is lost when the protein is denatured, although it returns upon renaturation or cooling below the glass transition.^6^,^7^ The isolated chromophore is virtually non-fluorescent in solution^8^ and *in vacuo*,^9^ but can become fluorescent when incorporated in rigid scaffolds such as metal–organic frameworks or other non-native protein environments.^10^–^13^ The lack of fluorescence from the free chromophores is widely accepted to be due to ultrafast isomerisation around the C^2^–C^3^–C^4^ bridge (Fig. 1) followed by efficient internal conversion taking excited state population back to the electronic ground state.^14^,^15^ When the molecular framework of the chromophore is held rigid by the protein or a rigid scaffold, isomerisation around the C–C–C bridge is impeded and fluorescence from the excited state is the primary decay channel.

Knowing that the environment of the chromophore plays a pivotal role in determining the spectral properties of GFP, which are important for imaging applications, led to numerous investigations to determine how structural modifications to the protein, such as changing single amino acid residues, can be used to tune the absorption or fluorescence properties.^4^,^16^ Recently, there has also been a great deal of interest in determining how the structure and environment of the isolated chromophore influences the absorption properties^17^,^18^ and dynamics.^19^ For example, variants of the isolated chromophore in which the hydroxyl group was moved from the *para* position to the *ortho* position were found to be weakly fluorescent in cyclohexane (*Φ* ≈ 0.003).^20^,^21^ The fluorescence was attributed to an intramolecular hydrogen-bond between the *ortho* OH group and the N atom of the imidazolinone group forming a seven-membered ring, which partly locked the molecular framework and restricted isomerisation around the C–C–C bridge. Recently, it was shown that the fluorescence quantum yield could be enhanced further (*Φ* ≈ 0.2) by introducing a five-membered ring to completely lock the C^3^–C^4^ single bond in the C–C–C bridge (Fig. 1).^22^ Chromophores in which the hydroxyl group has been moved from the *para* position to the *meta* position have also been found to be weakly fluorescent (*Φ* ≈ 0.002);^23^,^24^ in these cases, the fluorescence was attributed to changes in the electronic structure introducing a barrier along the isomerisation coordinate and thus decreasing the rate of isomerisation around the C–C–C bridge.

Upon irradiation with UV light, not only does GFP fluoresce, but it also has the ability to act as a light-induced electron donor,^25^,^26^ which is important for monitoring and manipulating redox processes in cells. The idea that the position of the hydroxy group and structural rigidity play key roles in controlling the fluorescence emission quantum yield, led us to ask how moving the O^–^ group and partly locking the molecular framework of the deprotonated chromophore anions might affect redox transformations.

A number of recent photoelectron spectroscopy and theoretical studies of the gas-phase isolated deprotonated chromophore anion, *p*-HBDI^–^, have shown that the vertical excitation energy of the first bright electronically excited ππ* state lies below the adiabatic threshold for photodetachment.^27^–^30^ The most accurate measurement of the adiabatic threshold for photodetachment has been made using vibrationally-resolved photoelectron spectroscopy and is found to be 2.73 eV.^31^ A separate photoelectron spectroscopy study has shown that adding water molecules to solvate the chromophore anion raises the adiabatic photodetachment threshold,^32^ supporting earlier computational studies showing that the hydrogen-bonding environment stabilises the first excited state against electron emission.^33^ Other computational studies have focused on the effect of the protein and specific interactions on the detachment energy.^34^,^35^ A femtosecond time-resolved study of the excited state dynamics of this first excited state found two characteristic timescales: 330 fs for isomerisation around the C–C–C bridge and 1.4 ps for subsequent internal conversion to the anion ground state,^36^ remarkably similar to the excited state dynamics of the isolated chromophore in solution measured by fluorescence up-conversion.^14^ A subsequent time-resolved study found the lifetime for competing autodetachment from the first excited state to be 30 ps,^37^ consistent with calculations.^38^ Just above the first detachment threshold, the second and third excited ππ* states are accessible^39^ and autodetachment from these states is found to compete with direct detachment.^27^,^30^,^40^

The aim of the work presented in this paper was to investigate how moving the O^–^ group from the *para* position to the *ortho* position, or adding an additional OH group at the *ortho* position to partly lock the molecular framework of the deprotonated chromophore anions, might affect the competition between electron detachment and internal conversion. Specifically, we recorded photoelectron spectra of *p*-HBDI, *o*-HBDI and *op*-DHBDI in their deprotonated anionic forms (Fig. 1) following photoexcitation with ultraviolet light in the range 400–230 nm. Using quantum chemistry calculations to guide our interpretation of the spectra, we find that our results support the idea that partial locking of the C–C–C bridge inhibits internal conversion of the deprotonated chromophore anion and we find that moving the position of the O^–^ group to the *ortho* position results in changes to the electronic structure that enhance internal conversion. These results have potential applications in tuning light-induced redox processes in biology.

## 2 Methodology

### 2.1 Chromophores

4-Hydroxybenzylidene-1,2-dimethylimidazolinone (*p*-HBDI) was prepared using reported procedures^41^ and 2-hydroxybenzylidene-1,2-dimethylimidazolinone (*o*-HBDI) was prepared using a modified version of a literature procedure.^42^ 2,4-Dihydroxybenzylidene-1,2-dimethylimidazolinone^22^ (*op*-DHBDI) was synthesised using an analogous route to those employed for *p*-HBDI and *o*-HBDI, *via* the dimethoxy analogue^43^ and deprotection, which was higher yielding than using 2,4-dihydroxybenzaldehyde directly.†

### 2.2 Photoelectron spectroscopy

Photoelectron spectra were recorded using our instrument that combines electrospray ionisation (ESI) and photoelectron velocity map imaging (VMI).^27^,^30^,^44^–^46^ Briefly, deprotonated anions were generated in the gas-phase by ESI of ∼0.1 mM solutions of chromophores in methanol with a few drops of aqueous ammonia. Chromophore anions were mass-selected by a quadrupole before passing into a hexapole ion trap. Anions were pulsed out of the trap and guided to the photodetachment region of a collinear VMI spectrometer where they interacted with laser pulses with wavelengths of 400 nm (3.10 eV), 346.1 nm (3.582 eV) and 230.7 nm (5.374 eV). The 400 nm pulses were generated by frequency-doubling the output of a commercial, amplified, Ti:sapphire femtosecond laser system operating at 1 kHz and the 346.1 nm and 230.7 nm pulses were generated by frequency doubling or tripling the output of a Nd:YAG pumped dye laser operating at 20 Hz. Following the anion–laser interaction, photodetached electrons were accelerated towards a position sensitive detector and imaged using a phosphor screen and CCD camera (Photek). Since anions and scattered laser light generate background counts, anion-only and laser-only images were subtracted from images recorded following the anion–laser interaction. The photoelectron images were inverted using the pBASEX method.^47^ The wavelength of the laser light was measured using a wavemeter and the energy scale of the detector was calibrated by recording the photoelectron spectrum of I^–^. The energy resolution of our instrument is <5% and the error in electron kinetic energy (eKE) is around 0.05 eV.

### 2.3 Calculations

In order to calculate VDEs of each chromophore, a combination of electron propagator theory^48^ (EPT) and time-dependent density functional theory^49^ (TD-DFT) calculations were employed using the Gaussian 09 computational chemistry software package.^50^ The geometries of the chromophore anions were optimised using B3LYP^51^,^52^ with the 6-311++G(3df,3pd)^53^ basis set. Vertical detachment energies from the minimum energy of the ground electronic state of the anion, S_0_, to the ground electronic state of the corresponding neutral radical, D_0_, were calculated using EPT applying the outer valence Green's function (OVGF) propagator^54^ with the 6-311++G(3df,3pd) basis set. This method has been found in earlier work to yield reliable VDEs.^30^,^45^,^46^ To calculate VDEs for higher lying electronically excited states of the neutral radical, D_1_–D_3_, TD-DFT calculations were carried out on the ground electronic state of the neutral radical at the minimum energy geometry of the ground electronic state of the anion using the CAM-B3LYP^55^/6-311++G(3df,3pd) method. These VEEs were added to the previously calculated vertical detachment energies to D_0_ to obtain the higher lying detachment energies. This combined methodology is referred to as EPT + TD-DFT.

In addition, the equation-of-motion coupled-cluster method with single and double excitations for the calculation of ionisation potentials^56^ (EOM-IP-CCSD) was employed together with the aug-cc-pVDZ^57^ basis set to calculate the first four VDEs. These calculations were carried out using the Q-Chem computational chemistry software package.^58^ The geometries of the deprotonated chromophore anions were minimised with Møller–Plesset perturbation theory of second order^59^ (MP2) and the aug-cc-pVDZ basis set using the Gaussian computational chemistry package.

VEEs of the excited singlet states of the anion were computed at the MP2 minima with the algebraic diagrammatic construction method to second order^60^,^61^ (ADC(2)) using the aug-cc-pVDZ basis set, employing the Turbomole computational chemistry software package.^62^ ADC(2) was chosen to be able to calculate higher electronically excited states reliably at a reasonable computational cost. Due to the number of diffuse functions in the basis set, a high number of continuum states are being calculated where that number of states depends on the selected basis set; however, the lower continuum states converge with increasing number of calculated states and smaller basis sets that exclude continuum states neglect the interaction of resonance states with the continuum.^63^

## 3 Results and discussion

### 3.1 Photoelectron spectra

**Fig. 2:**
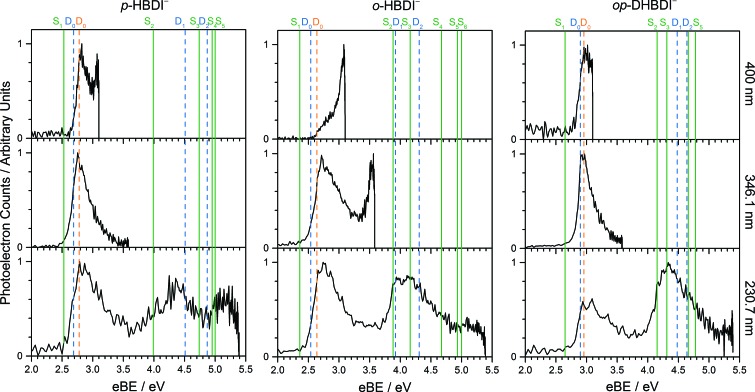
Photoelectron spectra of *p*-HBDI^–^ (left) *o*-HBDI^–^ (centre) and *op*-DHBDI^–^ (right) recorded at 400 nm (3.10 eV), 346.1 nm (3.582 eV) and 230.7 nm (5.374 eV). Individual spectra have been normalised to their maximum intensity. Vertical dashed orange and blue lines mark the first three vertical detachment energies, calculated using the EPT/6-311++G(3df,3pd) and EOM-IP-CCSD/aug-cc-pVDZ methods, respectively. Vertical green lines mark the VEEs of the anions calculated using the ADC(2)/aug-cc-pVDZ method (solid green).

**Fig. 3:**
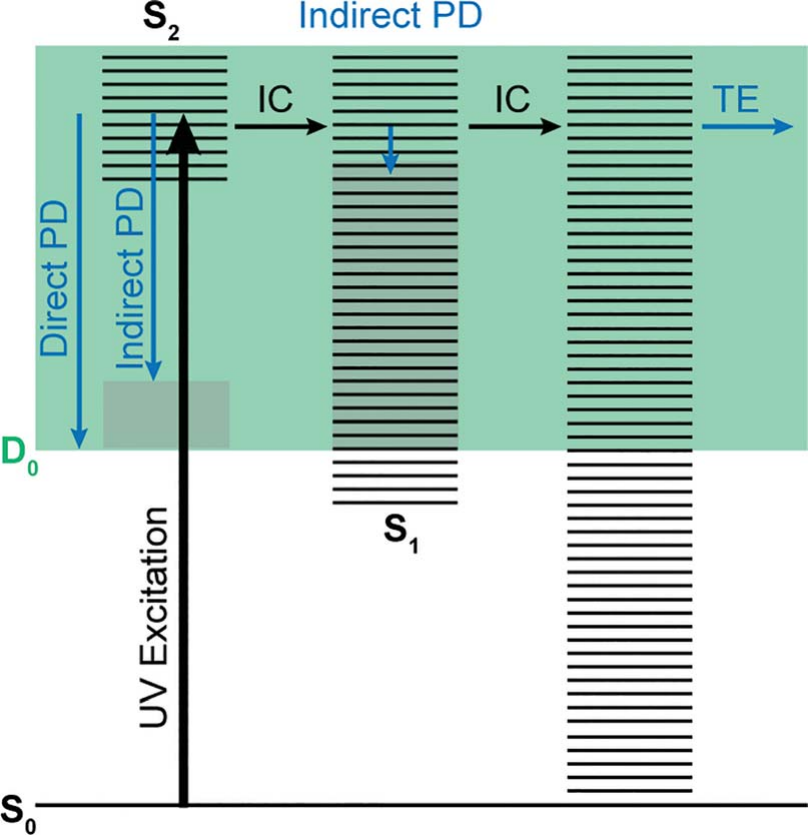
Jablonski diagram illustrating the electronic relaxation and emission processes following UV photoexcitation of the model GFP chromophores employed in this work. Horizontal black lines represent the vibrational levels of the excited electronic states and the solid green area represents the electron detachment continuum. Vertical blue arrows represent the eKE of direct and indirect photodetachment (PD) processes and the solid grey areas represent the vibrational energy left in the neutral radical after electron detachment (determined by the propensity for conservation of vibrational energy). The horizontal black arrows represent some of the possible internal conversion (IC) processes and the horizontal blue arrow represents electrons with eKE ∼0 eV following thermionic emission (TE) from vibrationally hot S_0_.

Photoelectron spectra of the deprotonated chromophores were recorded as a function of electron kinetic energy (eKE) and are presented as a function of electron binding energy, eBE = *hν* – eKE in Fig. 2. The electron emission processes contributing the photoelectron spectra are illustrated on a Jablonski diagram in Fig. 3.

#### 3.1.1 *p*-HBDI^–^

To benchmark our experiments, we recorded a photoelectron spectrum of *p*-HBDI^–^ at 350.0 nm (3.542 eV), which is similar to previously reported spectra recorded around this wavelength.^28^–^31^ It should be noted that this spectrum is significantly narrower than our own previously reported spectrum.

Spectra recorded at 400 nm (3.10 eV), 346.1 nm (3.582 eV) and 230.7 nm (5.374 eV) all have steep rising edges at around 2.7 eV, corresponding to direct detachment from S_0_ to D_0_ (Fig. 3), as reported by several experimental and theoretical groups.^27^–^31^ This is very close to the adiabatic detachment threshold which has been determined experimentally to be 2.73 eV and to be equivalent to the VDE.^31^ It is worth noting that the peak maxima are blue-shifted by around 0.1 eV from the calculated VDE, in agreement with calculated photoelectron spectra.^64^

The 400 nm photoelectron spectrum has a sharp feature at high eBE around 3.10 eV, very close to the photon energy of 3.10 eV. This high eBE (low eKE) feature has a profile that decays exponentially with eKE; this statistical eKE distribution is characteristic of thermionic emission from the hot ground electronic state of an anion, S_0_, following internal conversion from an excited electronic state (Fig. 3). The 346.1 nm photoelectron spectrum is dominated by direct detachment from S_0_ to D_0_ and there is no evidence of thermionic emission from the hot ground state. The 230.7 nm photoelectron spectrum is dominated by three, broad, unresolved features centered around 2.8 eV, 4.4 eV and 5.3 eV, which are attributed to detachment from S_0_ to D_0_, D_1_ and D_2_, respectively (Section 3.2). The broadening of the direct detachment peak on its high eBE edge in the 230.7 nm spectrum, compared to 346.1 nm spectrum, can be attributed to indirect detachment to D_0_ after population of high lying electronically excited states of the anion around 230.7 nm (5.374 eV) as illustrated in Fig. 3.

#### 3.1.2 *o*-HBDI^–^

Photoelectron spectra of *o*-HBDI^–^ recorded at 400 nm (3.10 eV), 346.1 nm (3.582 eV) and 230.7 nm (5.374 eV) all have rising edges at around 2.5 eV, corresponding to direct detachment from S_0_ to D_0_. This is ∼0.2 eV lower than in *p*-HBDI^–^, indicating that moving the O^–^ group from the *para* position to the *ortho* position lowers the detachment energy. This can be rationalised in terms of repulsion between the O^–^ group and the N-atom on the imidazolinone group pushing charge from the O^–^ group into the conjugated system, if we assume that the deprotonated chromophore anions are formed in the gas-phase in a *cis* geometry, *i.e.* the same as their parent neutral molecules. Calculations also show that in the *cis* geometry the conjugation over the bridge is weakened by the O^–^ group, which will also destabilise the molecular orbital (MO) from which the electron is removed (see Section 3.2). This is consistent with detailed investigations of fluorescent protein chromophores showing that the trend in detachment energy for the series of deprotonated chromophore anions *o*-HBDI^–^, *m*-HBDI^–^ and *p*-HBDI^–^, can be explained in terms of the resonance stabilisation of the anion.^65^,^66^ This is significant since it has been shown that the trend in redox potentials of a series of equivalent neutral chromophores is determined by the variation in ionisation energy since the solvent effects for structurally similar chromophores are similar.^67^

The 400 nm photoelectron spectrum is dominated by a sharp peak with eBE around the photon energy of 3.10 eV and whilst there is some contribution from direct detachment, it is much less significant. This suggests that moving the O^–^ group from the *para* position to the *ortho* position results in a more efficient internal conversion process funnelling excited state population back to the ground electronic state. The photoelectron spectrum recorded at 346.1 nm has a broad feature peaking around 2.7 eV and a sharp feature at high eBE (low eKE). Thus, unlike *p*-HBDI^–^, in *o*-HBDI^–^, internal conversion is still able to compete with direct detachment following excitation at 346.1 nm. The 230.7 nm photoelectron spectrum is dominated by two, broad, unresolved features centered around 2.6 eV and 4.2 eV, which are attributed to detachment from S_0_ to D_0_ and D_1_ (Section 3.2). There is also a third, less intense feature centered around 5.1 eV.

#### 3.1.3 *op*-DHBDI^–^

Photoelectron spectra of *op*-DHBDI^–^ recorded at 400 nm (3.10 eV), 346.1 nm (3.582 eV) and 230.7 nm (5.374 eV) all have steep rising edges at an eBE of around 2.9 eV, corresponding to direct detachment from S_0_ to D_0_. The photoelectron spectra of *op*-DHBDI^–^ are remarkably similar to those of *p*-HBDI^–^, but shifted by ∼0.2 eV to higher eBE. The *op*-DHBDI chromophore has two deprotonation sites. Since the *ortho*-OH group stabilises the anion by forming a hydrogen bond with the nearby N atom of the imidazolinone unit, we conclude that the *op*-DHBDI^–^ is deprotonated predominantly at the *para* site. From the MOs (see Sec. 3.2), the O-atom of the *ortho*-OH group does not play a role in the ring conjugation, so it acts as an electron withdrawing group and stabilises the MO from which the electron is removed.

In contrast to *p*-HBDI^–^ and *o*-HBDI^–^, the spectrum of *op*-HBDI^–^ at 400 nm is dominated by direct detachment, although there is a slight increase in photoelectron counts at high eBE (low eKE) suggesting that there is a small contribution from thermionic emission from the ground electronic state. This seems most likely to be the result of hydrogen bonding between the *ortho*-OH group and the N atom of the imidazolinone unit restricting torsional motion around the central C–C–C bridge, which is required for efficient internal conversion back to the ground electronic state.^14^,^15^,^36^,^38^ The 230.7 nm photoelectron spectrum is dominated by two, broad, unresolved features centered around 3.0 eV and 4.4 eV, which are attributed to detachment from S_0_ to D_0_ and D_1_, respectively (Section 3.2). As with *p*-HBDI^–^, broadening of the direct detachment peak on its high eBE edge in the 230.7 nm spectrum, compared to the 346.1 nm spectrum, can be attributed to indirect detachment to D_0_ following population of an electronically excited state of the anion at 230.7 nm (5.374 eV). It is interesting to note that the band associated with detachment from S_0_ to D_1_ is more intense than the band associated with detachment from S_0_ to D_0_, which is opposite to the relative intensities observed in the photoelectron spectra of the *p*-HBDI^–^ and *o*-HBDI^–^ chromophores.

### 3.2 Computational results

**Fig. 4:**
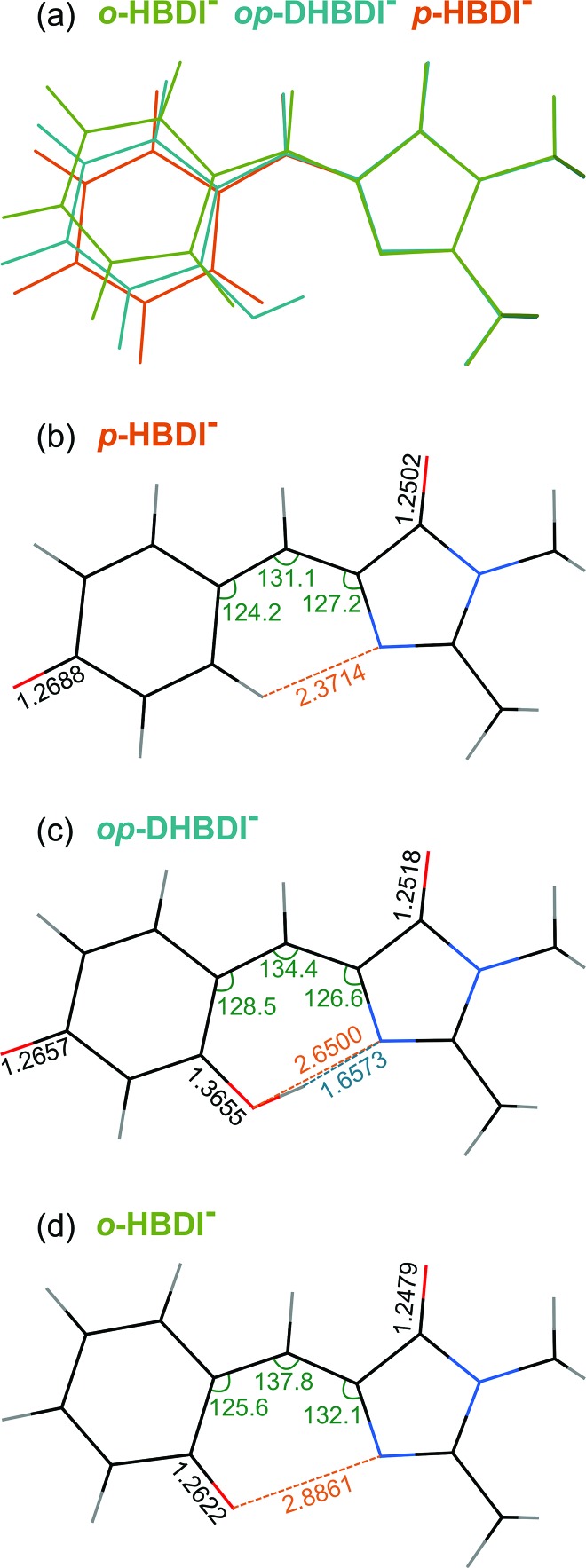
(a) Overlay of the optimised structures of *p*-HBDI^–^, *op*-DHBDI^–^ and *cis o*-HBDI^–^. (b)–(d) Geometries of the anions with selected geometrical data.

All three molecules are found to have stable planar geometries calculated at the MP2/aug-cc-pVDZ level of theory (Fig. 4).† The effect of adding the substituents at the *ortho* position is to open up the bridge. In *p*-HBDI^–^ the C–C–C angle of the bridge joining the two rings is 131.1°, in *op*-DHBDI^–^ it is 134.4° and in *o*-HBDI^–^ 137.8°. The planar geometry of *cis o*-HBDI^–^ is, however, not a minimum which is actually found to be with a C^2^–C^3^–C^4^–C^5^ torsion angle at the bridge of 23.5° (using the numbering in Fig. 1), which allows the bridge angle to close up to 133.3°. This is a very shallow minimum of 0.017 eV, indicating that the conjugation on the bridge has been weakened making it more susceptible to rotation.

It should be noted that the *o*-HBDI^–^ chromophore has two stable conformations, with the O^–^ group either *cis* or *trans* to the imidazolinone nitrogen (atom 1 in Fig. 1). In the parent neutral molecule, the *cis* chromophore is the minimum energy geometry, because it is stabilised by a hydrogen bond between the OH group and the imidizolinone nitrogen.^20^,^68^ A relaxed scan on the anion around the torsion angle starting in the *cis* geometry shows a barrier height of approximately 0.4 eV to the isomerisation, which is high compared to the energy available in the formation of the ion. Thus it seems likely that the chromophore is formed in the *cis* geometry during the ESI process even though the global minimum for this molecule is found to be the *trans* geometry by approximately 0.4 eV.† Our analysis of the photoelectron spectra supports this (Section 3.3), and for the rest of the discussion all results for *o*-HBDI refer to the *cis* conformer, unless stated otherwise.

In *op*-DHBDI^–^, the hydrogen bond between the *ortho*-OH group and the imidizolinone nitrogen stabilises the *cis* geometry of the anion and this is the minimum energy structure. Relaxed scans around the torsion support the reduced flexibility of the bridge in *op*-DHBDI^–^ as the barrier to rotation from *cis* to *trans* in this molecule is approximately 1.4 eV.†

**Fig. 5:**
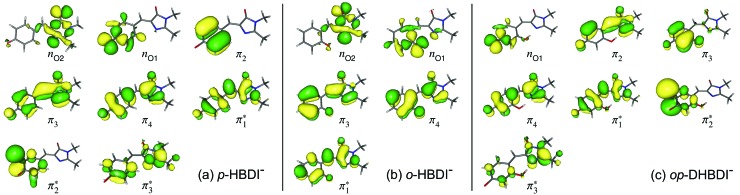
Hartree–Fock (HF) orbitals of (a) *p*-HBDI^–^, (b) *o*-HBDI^–^ and (c) *op*-DHBDI^–^ used in the ADC(2)/aug-cc-pVDZ calculations. The HOMO is π_4_ and the LUMO is 
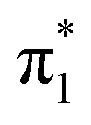
. The HF orbitals that were employed in the EOM-IP-CCSD calculations are very similar and therefore not shown.

The VDEs calculated using both the EPT + TD-DFT and EOM-IP-CCSD methods are listed in Table 1, together with the characters of the EOM-IP-CCSD states. VDEs for *trans o*-HBDI^–^ are very similar to those for the *cis* conformer.† The orbitals are presented in Fig. 5. The EOM-IP-CCSD calculated values for D_0_–D_2_ are indicated in Fig. 2, together with the EPT calculated value for D_0_ as a comparison. The higher lying ionisations calculated at the EPT + TD-DFT values are similar to the higher level of theory EOM-IP-CCSD results and are omitted from the plots for clarity. The VDEs to D_0_ for *p*-HBDI^–^, *o*-HBDI^–^ and *op*-DHBDI^–^ are 2.69 eV (2.78 eV), 2.54 eV (2.64 eV) and 2.90 eV (2.96 eV), calculated using the EOM-IP-CCSD (EPT + TDDFT) method. These calculated values follow the same trend as the experimental photoelectron spectra, with the VDEs increasing in the order, *o*-DHBDI^–^ < *p*-HBDI^–^ < *op*-HBDI^–^. For all the chromophores, the D_0_ state corresponds to a hole in a π orbital which is the HOMO.

**Table 1:** VDEs (in eV) for all anions calculated using the EPT + TD-DFT/6-311++G(3df,3pd) and EOM-IP-CCSD/aug-cc-pVDZ methods for the first four ionisation continua. The EPT value for D_0_ and all the EOM-IP-CCSD VDEs are plotted on the photoelectron spectra in Fig. 2. For EOM-IP-CCSD VDEs, the state character is given in parentheses to indicate the orbital in which the electron hole is present. The corresponding Hartree–Fock orbitals are presented in Fig. 5

Anion	State	EPT + TD-DFT	EOM-IP-CCSD	
*p*-HBDI^–^	D_0_	2.78	2.69	(π_4_)
	D_1_	4.34	4.51	(n_O1_)
	D_2_	4.85	4.87	(π_3_)
	D_3_	5.40	5.39	(π_2_)
*cis o*-HBDI^–^	D_0_	2.64	2.54	(π_4_)
	D_1_	3.97	3.92	(n_O1_)
	D_2_	4.31	4.20	(π_3_)
	D_3_	5.24	5.33	(n_O2_)
*op*-DHBDI^–^	D_0_	2.96	2.90	(π_4_)
	D_1_	4.46	4.48	(π_3_)
	D_2_	4.50	4.64	(n_O1_)
	D_3_	5.14	5.16	(π_2_)

To provide a benchmark of the calculated values, the VDE of *p*-HBDI^–^ compares well to the experimental value determined by high-resolution vibrationally-resolved photoelectron spectroscopy of 2.73 eV (ref. 31) and the value calculated at the XMCQDPT2/(aug)-cc-pVTZ level of theory of 2.68 eV.^29^,^39^ The VDEs of the first two excited radical states D_1_ and D_2_ in Table 1 are slightly higher than the XMCQDPT2/(aug)-cc-pVTZ values of 3.97 eV and 4.40 eV; however, the characters are the same, with D_1_ having a hole in the n_O_ orbital and D_2_ in a π orbital.^39^

As in *p*-HBDI^–^, D_1_ of *o*-HBDI^–^ is of n_O_ character, whereas D_1_ for *op*-DHBDI^–^ has a hole in a π orbital. This explains the change in the relative intensities of the D_0_ and D_1_ bands observed in the 230.7 nm photoelectron spectrum of *op*-DHBDI^–^ compared to the other two analogues since the transition probability (defined as the square of the norm of the Dyson orbital) between a continuum state of π character with a ππ* excited state would be greater than that for an nπ* excited state, especially if the same π orbital is involved. For all three chromophores, D_1_ and D_2_ are accessible at 230.7 nm; however, the VDEs for D_1_ are higher in energy than the 346.1 nm and 400 nm photon energies. Thus, at 346.1 nm and 400 nm, direct detachment to D_1_ and D_2_ is not possible and the D_1_ and D_2_ continua would only be accessible by indirect detachment from high-lying electronically excited states of the anions.

Relevant ADC(2)/aug-cc-pVDZ VEEs and their corresponding oscillator strengths and configurations are presented in Table 2 and plotted in Fig. 2. VEEs for *trans o*-HBDI^–^ are very similar to those for the *cis* conformer.† These VEEs are for the electronically excited states with the strongest oscillator strengths and, thus, realistically accessible. All of these states, with one exception, are of ππ* character. The details of all calculated excited states for all three chromophores, including other ππ*, nπ* and continuum states, can be found in the ESI.† It is difficult to directly compare the ADC(2) excited states of *p*-HBDI to those calculated at the XMCQDPT2/(aug)-cc-pVTZ level^69^ as these short-lived states are very sensitive to basis set and method used. In both cases, however, a set of accessible (*i.e.* non-negligible oscillator strength) ππ* states are found at around 4 eV, which have shape resonance character with respect to the D_0_ continuum.

**Table 2:** VEEs (in eV) of all anions calculated with ADC(2)/aug-cc-pVDZ with main configurations and corresponding oscillator strengths, *f*. The orbitals involved in the excitations are shown in Fig. 5

State	*p*-HBDI^–^			*cis o*-HBDI^–^			*op*-DHBDI^–^		
	*E*/eV	Config.	*f*	*E*/eV	Config.	*f*	*E*/eV	Config.	*f*
S_1_	2.53	π_4_ → 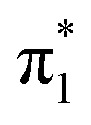	0.9483	2.36	π_4_ → 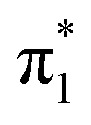	0.4429	2.65	π_4_ → 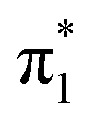	0.9848
S_2_	3.99	π_4_ → 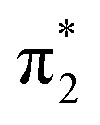	0.0445	3.88	π_3_ → 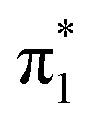	0.1743	4.15	π_4_ → 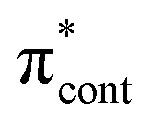	0.0176
S_3_	4.74	π_2_ → 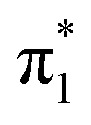	0.0229	4.16	π_4_ → 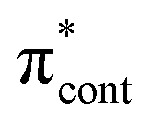	0.0261	4.31	π_4_ → 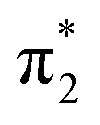 /π_4_ → 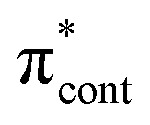	0.0390
S_4_	4.95	π_4_ → 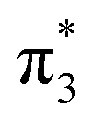	0.0735	4.67	π_4_ → 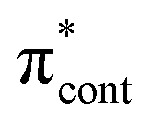	0.0206	4.66	π_4_ → 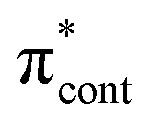 /π_4_ → 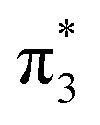	0.0666
S_5_	5.00	n_O2_ → 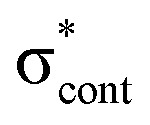	0.0114	4.93	π_4_ → 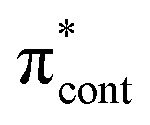	0.2487	4.78	π_4_ → 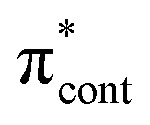	0.0236
S_6_				5.00	π_2_ → 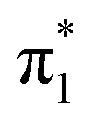 /π_4_ → 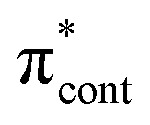	0.4115			

**Fig. 6:**
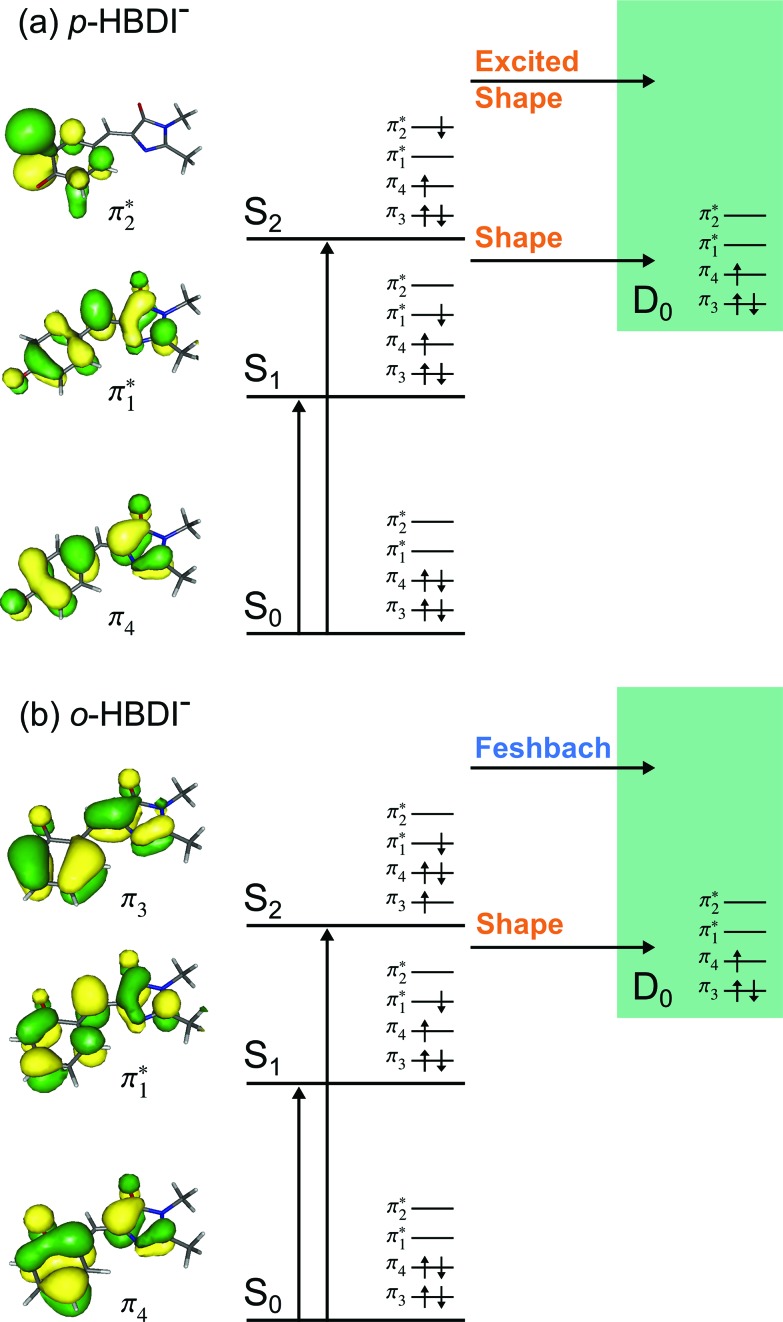
Energy level diagram of (a) *p*-HBDI^–^ and (b) *o*-HBDI^–^ in which possible excitations and subsequent detachment processes are indicated (note that it is only the vibrationally excited states of S_1_ lying in the D_0_ continuum that have shape resonance character). S_1_ and S_2_ refer to the first two excited states selected in Table 2. The HOMO is π_4_ and the LUMO is 
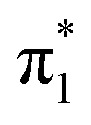
. Alongside are the Hartree–Fock molecular orbitals that are involved in the excitations. For *op*-DHBDI^–^, a similar scheme applies as for *p*-HBDI^–^ except the excited state resonance is S_3_ (Table 2).

The VEEs and their corresponding configurations for *p*-HBDI^–^ and *op*-DHBDI^–^ show similar trends. In both chromophores, the first excited state (ππ*) is very bright with oscillator strengths close to unity, whereas all other states have very low oscillator strengths. Additionally, the energy levels of *p*-HBDI^–^ and *op*-DHBDI^–^ are very similar to one another. In contrast, the S_1_ state of *o*-HBDI^–^ has a lower oscillator strength of 0.4429 but on the other hand, the S_2_ state has a higher oscillator strength than the S_2_ states in *p*-HBDI^–^ and *op*-DHBDI^–^. The S_2_ state of *o*-HBDI^–^ is described by a transition from the HOMO–1 into a π* orbital delocalised across the whole chromophore, whereas for *p*-HBDI^–^ and *op*-DHBDI^–^, the S_2_ state corresponds to the promotion of an electron from the HOMO into a π* orbital located at the benzyl moiety. The corresponding MOs are presented in Fig. 6 and in the ESI.† Additionally in *o*-HBDI^–^, there are two higher excited states at 4.93 and 5.00 eV with reasonably high oscillator strengths. The state at 4.93 eV corresponds to a transition from a bound π orbital to a π* orbital with continuum character and the state at 5.00 eV has some of the same character. These states could play a role in the electron emission process in the 230.7 nm photoelectron spectra.

### 3.3 Competition between detachment and internal conversion

Fig. 6 shows electronic energy level diagrams for *p*-HBDI^–^/*op*-DHBDI^–^ and *o*-HBDI^–^, together with relevant molecular orbitals. Included are the states that are most important in the dynamics of detachment to the D_0_ continuum, which are the first two excited states with ππ* character, referred to as S_1_ and S_2_. Possible excitations and their corresponding detachment processes are indicated.

In the case of *p*-HBDI^–^ (Fig. 6a), detachment from either electronically excited state requires the removal of one electron to reach D_0_. Hence, they have shape resonance character and autodetachment from both states is extremely fast. Due to its very large oscillator strength (0.9483), the transition from S_0_ to S_1_ is strongly resonant. The photon energy at 400 nm (3.10 eV) is low enough to excite S_1_ significantly, as illustrated by the action absorption spectrum of this chromophore.^39^ Population from S_1_ can then rapidly internally convert to highly excited vibrational levels of S_0_. The internal vibrational energy in S_0_ is high enough for thermionic emission to occur, which gives rise to the high eBE (low eKE) peak in the 400 nm spectrum. Such processes have been previously observed for *p*-HBDI^–^.^28^,^30^,^40^ and photoactive yellow protein chromophores.^45^,^46^,^70^

The S_2_ state lies at higher energy (3.99 eV), so it can only be excited significantly at the shortest laser wavelength used in our experiments. For this reason, it is unlikely that resonant processes from S_2_ to D_0_ impact the electron emission dynamics in the 400 nm or the shape of the D_0_ band. However, at 230.7 nm (5.374 eV) the photon energy is high enough to excite S_2_, whose minimum structure is significantly different from the S_0_ minimum. Moreover, the S_2_ state has excited shape resonance character with respect to the D_0_ continuum, which results in rapid indirect detachment and vibrational autodetachment from S_2_ and accounts for the broadening of the D_0_ band on the high eBE side.^69^ Such broadening is also observed on the high eBE side of the 346.1 nm photoelectron spectrum following excitation of low vibrational states of S_2_.^69^

The energy level diagram for *op*-DHBDI^–^ is essentially identical to that of *p*-HBDI^–^, only shifted to slightly higher energies. At 400 nm, the photon energy is closer to the VDE of S_1_, so at first sight it would seem likely that indirect detachment might play a more important role than direct detachment in electron emission from *op*-DHBDI^–^ compared to *p*-HBDI^–^. Since this is not the case and it appears that there is less thermionic emission from *op*-DHBDI^–^ than *p*-HBDI^–^ following excitation at 400 nm (Fig. 2), we conclude that the S_1_/S_0_ conical intersection is less accessible in *op*-DHBDI^–^ than in *p*-HBDI^–^. This can be rationalised in terms of the hydrogen-bonding between the OH group in the *ortho* position and the N atom on the imidazolinone moiety restricting torsional motions around the C–C–C bridge, which are known to be important in directing the excited state population to the S_1_/S_0_ conical intersection. Importantly, these observations also support solution phase studies of neutral *o*-HBDI and *op*-DHBDI chromophores, in which the formation of a hydrogen-bond between the *ortho*-OH and the N of the imidazolinone group has been shown to lock the molecular framework sufficiently to enhance the fluorescence quantum yield from the first excited state by impeding internal conversion.^21^,^22^,^42^ Similar restrictions to torsional motions are also responsible for the enhanced fluorescence yield of chromophores locked inside rigid scaffolds.^10^–^13^

Due to the differences in the oscillator strengths and state characters for *o*-HBDI^–^, we obtain a very different picture for the dynamics, as illustrated in Fig. 6b. The S_2_ state has Feshbach resonance character with respect to D_0_ (detachment is a two-electron process) indicating that it is only weakly coupled to the continuum. Hence, autodetachment from S_2_ will be slow. Since there is the possibility of significantly populating S_2_ due to its relatively high oscillator strength (0.1743) compared with *p*-HBDI^–^ and *op*-DHBDI^–^, and subsequent autodetachment is slow, we would expect internal conversion to lower lying electronic states to dominate. This explains why the peak at high eBE (low eKE), which can be attributed to thermionic emission from S_0_ or autodetachment from high lying energy levels of S_1_, appears in the 346.1 nm photoelectron spectrum of *o*-HBDI^–^ but not in the 346.1 nm photoelectron spectra of *p*-HBDI^–^ or *op*-DHBDI^–^. Also, at 400 nm, the photoelectron spectrum of *o*-HBDI^–^ is completely dominated by a high eBE (low eKE) thermionic emission peak. It seems very unlikely that this could be attributed to internal conversion from S_2_ since at this photon energy it is unlikely that even the lowest vibrational levels of S_2_ would be accessible. It seems far more likely that excitation of high lying vibrational states of S_1_ results in more efficient internal conversion to S_0_ in *o*-HBDI^–^ compared to *p*-HBDI^–^ or *op*-DHBDI^–^. This can be rationalised in terms of the repulsion between the O^–^ group at the *ortho* position and the N atom on the imidazolinone group enhancing torsional motions around the C–C–C bridge as discussed above (Section 3.2); and supports our suggestion that the *o*-HBDI^–^ chromophore is formed predominantly in its *cis* geometry in the gas phase.

## 4 Conclusions

From the combination of experimental and computational results, we have advanced our understanding of how the OH group affects the photodynamics of the model GFP chromophore. For longer wavelengths of light closer to the visible range, we have shown that by moving the O^–^ group from the *para* position to the *ortho* position, we have an effective way of enhancing internal conversion back to the S_0_ ground state of the chromophore as a decay pathway which is highly competitive with electron emission. Conversely, introducing an OH group at the *ortho* position in addition to the *para* O^–^ group allows us to enhance the electron emission process by impeding internal conversion. This dynamical control is driven by changes to the molecular and nuclear structure that control torsional motions around the C–C–C bridge in the chromophore. We also find that moving the O^–^ group from the *para* position to the *ortho* position results in a decrease in photon energy required for electron detachment by ∼0.2 eV whereas introducing an OH group at the *ortho* position in addition to the *para* O^–^ group increases the photon energy required for electron detachment by ∼0.2 eV. This type of chemical control allows us to control the wavelength of light required to initiate the redox processes in GFP chromophores. The information obtained for processes at higher photon energies remains more general. Although this region may be of less interest for the fluorescence properties of GFP chromophores, it could be more important in the control of light induced electron driven processes. For the highest energy photons employed in this study (230.7 nm), we found that many additional electronically excited states of the anion and neutral radical become accessible for all chromophores. The photodynamics are similar for all the chromophores with the O^–^ and OH groups simply acting as tuning substituents. The oscillator strengths of the higher lying states of the *o*-HBDI^–^ chromophore are brighter than in the other two analogues, so may play a more important role in the electron emission processes at shorter wavelengths. The next step is to follow the excited state dynamics in time-resolved experiments, which we plan to do.

In summary, we have shown that the electron emission and competing processes can be controlled by modifying the substitution pattern in the phenoxide moiety. We propose that the ability to control these processes will play an important role in tuning light-induced redox processes of the biologically and technologically important family of fluorescent proteins derived from GFP.

## Supplementary Material

Supplementary information
