# Supplementary material for: ortho and para chromophores of green fluorescent protein: controlling electron emission and internal conversion
Source: Chem Sci. 2016 Nov 7;8(2):1621–30. doi: 10.1039/c6sc03833f (PMC5933426; doi:10.1039/c6sc03833f)
Supplement: Supplementary file 1 [file SC-008-C6SC03833F-s001.pdf]

Electronic Supplementary Material (ESI) for Chemical Science.
This journal is © The Royal Society of Chemistry 2016

Electronic Supplementary Information

Ortho and *para* chromophores of green fluorescent protein: controlling electron emission and internal conversion

Conor McLaughlin,[a] Mariana Assmann,[a] Michael A. Parkes,[a] Joanne Woodhouse,[a] Ross Lewin,[a] Helen C. Hailes,[a] Graham A. Worth,[a] and Helen H. Fielding[a*]

[a]Department of Chemistry, University College London, 20 Gordon Street, London WC1H 0AJ, UK

1 Synthesis of the reagents

All reagents were used as supplied without further purification unless stated otherwise. Anhydrous solvents were either purchased from Sigma Aldrich or Thermo Fisher, or were dried by treatment with activated 3 Å molecular sieves.[1] Thin layer chromatography (TLCs) were performed on silica gel plates with a fluorescent indicator, flash chromatography was performed using silica gel with a 40-63 μm pore size, and petroleum ether used in silica chromatography was the 40-60 fraction. NMR spectra were recorded on Bruker spectrometers AMX300, Avance 500 and Avance III 600. Chemical shifts (in ppm) are given relative to tetramethylsilane and referenced to residual protonated solvent. Coupling constants (J) are measured in Hertz (Hz) and multiplicities for ^{1}H NMR coupling are shown as s (singlet), d (doublet), t (triplet), and m (multiplet). Mass spectrometry analyses were performed at the UCL Chemistry Mass Spectrometry Facility using a Finnigan MAT 900XP mass spectrometer. Melting points were recorded on a Stuart SMP10 or SMP11 melting point apparatus and are uncorrected.

4-Hydroxybenzylidene-1,2-dimethylimidazolinone (*p*-HBDI) was prepared using reported procedures.[2] It was purified by recrystallisation (ethanol). 4-(2-Methoxybenzylidene)-1,2-dimethyl-1*H*-imidazol-5(4*H*)-one was synthesized as previously described.[3]

(*Z*)-4-(2-Hydroxybenzylidene)-1,2-dimethyl-1*H*-imidazol-5(4H)-one[3] (*o*-HBDI)

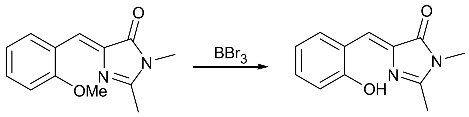

Boron tribromide (43.0 mL of a 1 M solution in CH_2Cl_2; 43.0 mmol) was added dropwise to a solution of 4-(2-methoxybenzylidene)-1,2-dimethyl-1*H*-imidazol-5(4H)-one[3] (1.837 g, 7.98 mmol)

in CH_2Cl_2 (50 mL) under argon at 0 °C, resulting in the formation of a deep red solution and precipitate. The reaction was stirred at room temperature for 18 h, water (50 mL) was added dropwise, and the organic phase dried ($MgSO_4$) and purified by silica column chromatography (ethyl acetate/hexane, 1:1) to yield *o*-HBDI[3] as a yellow solid (0.720 g, 42%); M.p. 227-233 °C (decomposes); υ_{max} (neat) 2926, 1705, 1647, 1558, 1419 cm^{1}; ^{1}H NMR (600 MHz; $CDCl_3$) δ 13.74 (1H, br s, OH), 7.33-7.36 (1H, m, 4-H), 7.30 (1H, dd, J = 8.0 and 1.5 Hz, 6-H), 7.18 (1H, s, =C*H*Ar), 6.95 (1H, dd, J = 8.0 and 0.5 Hz, 3-H), 6.90 (1H, t, J = 8.0 Hz, 5-H), 3.23 (3H, s, NCH_3), 2.39 (3H, s, N=CCH_3); ^{13}C NMR (151 MHz; $CDCl_3$) δ 168.1, 158.7, 157.6, 136.6, 134.3, 132.9, 130.5, 119.8, 119.6, 119.4, 27.0, 15.4; *m/z* HRMS (ES+) found $[MH]^+$ 217.0979; $C_{12}H_{13}N_2O_2$ requires 217.0977.

(*Z*)-4-(2,4-Dimethoxybenzylidene)-2-methyloxazol-5(4H)-one[4]

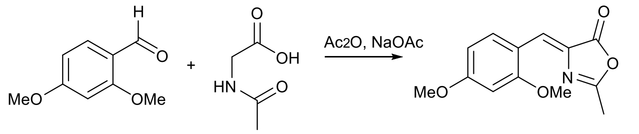

2,4-Dimethoxy benzaldehyde (14.13 g, 85.0 mmol), *N*-acetyl glycine (10.01 g, 85.5 mmol) and sodium acetate (7.01 g, 85.5 mmol) in were heated at 90 °C in acetic anhydride (100 mL) for 4 h, after which the mixture was cooled to room temperature, and left to stand for 18 h. The precipitate formed was collected by filtration and further precipitate was afforded by the addition of water to the filtrate. The combined precipitates were washed with water (2 x 30 mL) and dried to yield 4-(2,4-dimethoxybenzylidene)-2-methyloxazol-5(4H)-one[4] as orange needles (6.13 g, 29%). M.p. 160-164 °C (lit. 164-165 °C);[4] υ_{max} (neat) 1651, 1571, 1419 cm^{1}; ^{1}H NMR (300 MHz; $CDCl_3$) δ 8.65 (1H, d, J = 8.8 Hz, 6-H), 7.68 (1H, s, =C*H*Ar), 6.59 (1H, dd J = 8.8 and 2.0 Hz, 5-H), 6.42 (1H, d, J = 2.0 Hz, 3-H), 3.87 (6H, s, 2 x OMe), 2.37 (3H, s, N=CCH_3); ^{13}C NMR (101 MHz; DMSO-d_6) δ 167.8, 165.0, 163.9, 160.6, 133.3, 129.2, 123.2, 114.4, 107.1, 97.9, 56.1, 55.7, 15.3; *m/z* HRMS (ES+) found $[MH]^+$ 248.0926; $C_{13}H_{14}NO_4$ requires 248.0923.

(*Z*)-4-(2,4-Dimethoxybenzylidene)-1,2-dimethyl-1*H*-imidazol-5(4H)-one[5]

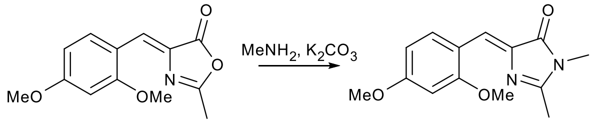

4-(2,4-Dimethoxybenzylidene)-2-methyloxazol-5(4H)-one (6.13 g, 24.8 mmol), potassium carbonate (3.78 g, 27.4 mmol) and methylamine (5.4 mL of a 40% aqueous solution) in ethanol (80 mL) were heated at reflux for 4 h. The reaction was then cooled to 0 °C and the resulting yellow precipitate formed was collected by filtration to yield (*Z*)-4-(2,4-dimethoxybenzylidene)-1,2-dimethyl-1*H*-imidazol-5(4H)-one (4.14 g, 64%) as a yellow solid. M.p. 192 °C (decomposes); υ_{max} (neat) 1694, 1628, 1593 cm^{1}; ^{1}H NMR (600 MHz; DMSO-d_6) δ 8.73 (1H, d, J = 8.8 Hz, 6-H), 7.25 (1H, s, =C*H*Ar), 6.66 (1H, dd, J = 8.8 and 2.2 Hz, 5-H), 6.61 (1H, d, J = 2.2 Hz, 3-H), 3.88 (3H, s, OMe), 3.83 (3H, s, OMe), 3.08 (3H, s, NCH_3), 2.33 (3H, s, N=CCH_3); *m/z* HRMS (ES+) found $[MH]^+$ 261.1242; $C_{14}H_{17}N_2O_3$ requires 261.1239.

(*Z*)-4-(2,4-Dihydroxybenzylidene)-1,2-dimethyl-1*H*-imidazol-5(4H)-one[6] (*op*-DHBDI)

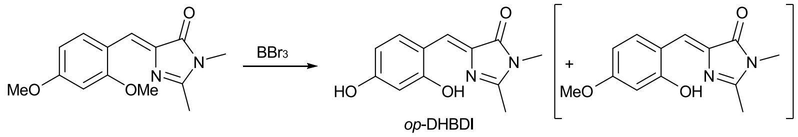

Boron tribromide (79.0 mL of a 1 M solution in CH_2Cl_2; 79.0 mmol) was added dropwise to a solution of (*Z*)-4-(2,4-dimethoxybenzylidene)-1,2-dimethyl-1*H*-imidazol-5(4H)-one (3.70 g, 14.2 mmol) in CH_2Cl_2 (140 mL) under argon at 0 °C, resulting in the formation of a deep red solution and precipitate. The reaction was stirred at room temperature for 18 h, water (140 mL) was added dropwise, and the organic phase dried ($MgSO_4$) and purified by silica column chromatography (ethyl acetate/petroleum ether, 1:2, R_f 0.12) to yield *op*-DHBDI (0.700 g, 21%)[6] as a yellow solid [(*Z*)-4-(2-hydroxy-4-methoxybenzylidene)-1,2-dimethyl-1*H*-imidazol-5(4H)-one was also formed (0.670 g, 19%)]; M.p. 150 °C (decomposes); υ_{max} (neat) 3303, 2922, 1689, 1643, 1597, 1564, 1503 cm^1; ^{1}H NMR (600 MHz; DMSO-d_6) δ 12.42 (1H, br s, OH), 10.20 (1H, br s, OH), 7.87 (1H, d, J = 8.6 Hz, 6-H), 7.13 (1H, s, =C*H*Ar), 6.32 (1H, dd, J = 8.6, 2.4 Hz, 5-H), 6.27 (1H, d, J = 2.4 Hz, 3-H), 3.10 (3H, s, NCH_3), 2.34 (3H, s, N=CCH_3); ^{13}C NMR (151 MHz; DMSO-d_6) δ 168.3, 162.5, 159.9, 159.2, 136.3, 132.0, 124.7, 112.4, 108.3, 103.2, 26.4, 15.1; *m/z* HRMS (ES+) found $[MH]^+$ 233.0925; $C_{12}H_{13}N_2O_3$ requires 233.0921.

2 NMR spectra

(*Z*)-4-(2-Hydroxybenzylidene)-1,2-dimethyl-1*H*-imidazol-5(4H)-one[3] (*o*-HBDI)

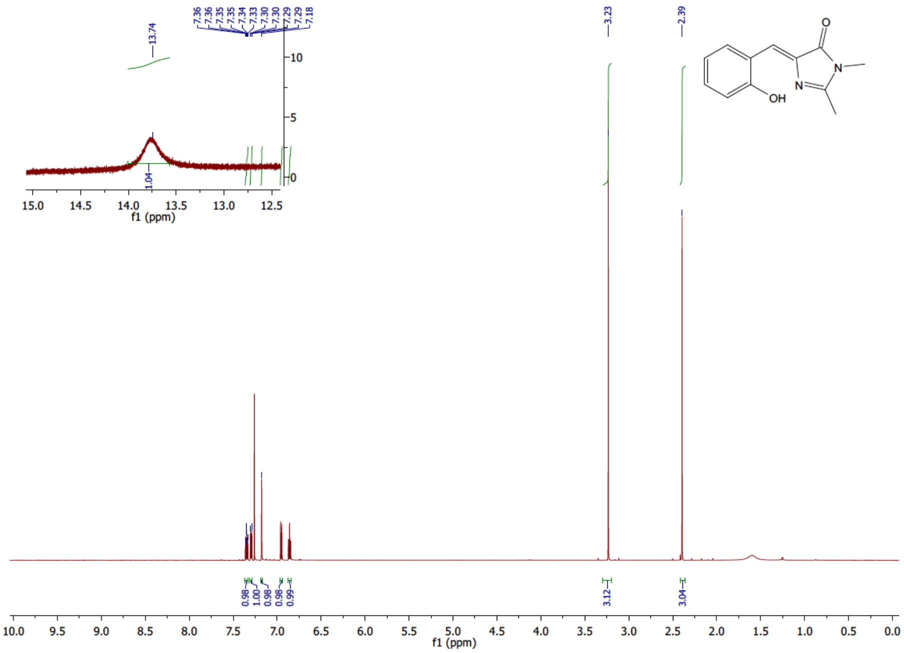

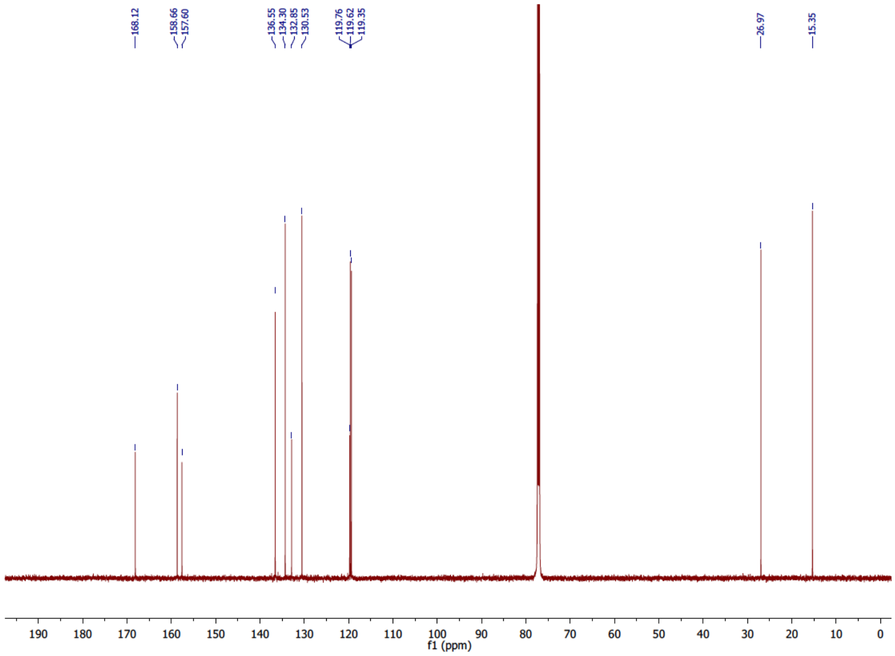

(*Z*)-4-(2,4-Dimethoxybenzylidene)-1,2-dimethyl-1*H*-imidazol-5(4H)-one[5]

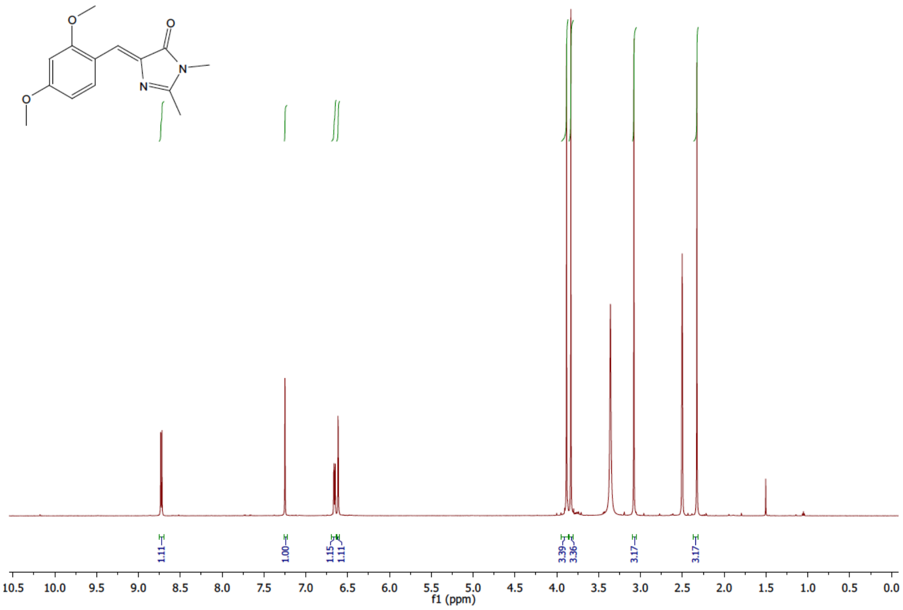

(*Z*)-4-(2,4-Dihydroxybenzylidene)-1,2-dimethyl-1*H*-imidazol-5(4H)-one[6] (*op*-DHBDI)

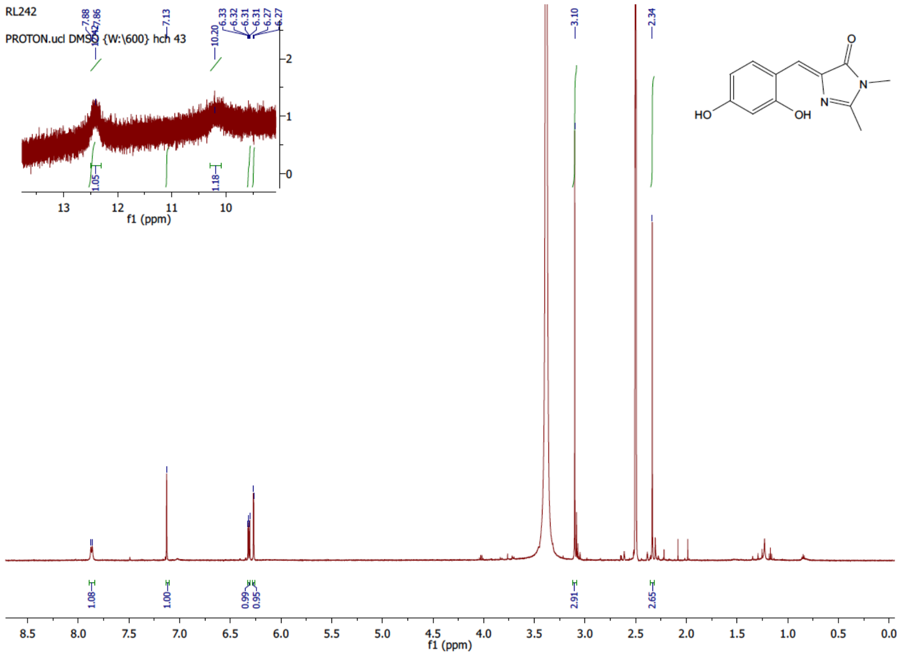

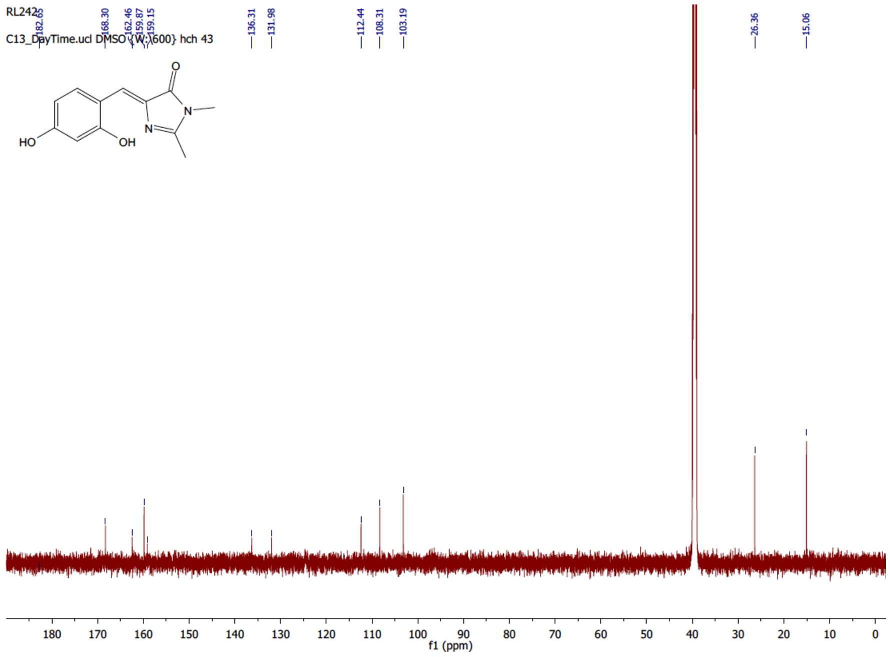

3 Scans along the bridge and CCOH torsion in *o*-HBDI$^-$ and *op*-DHBDI$^-$

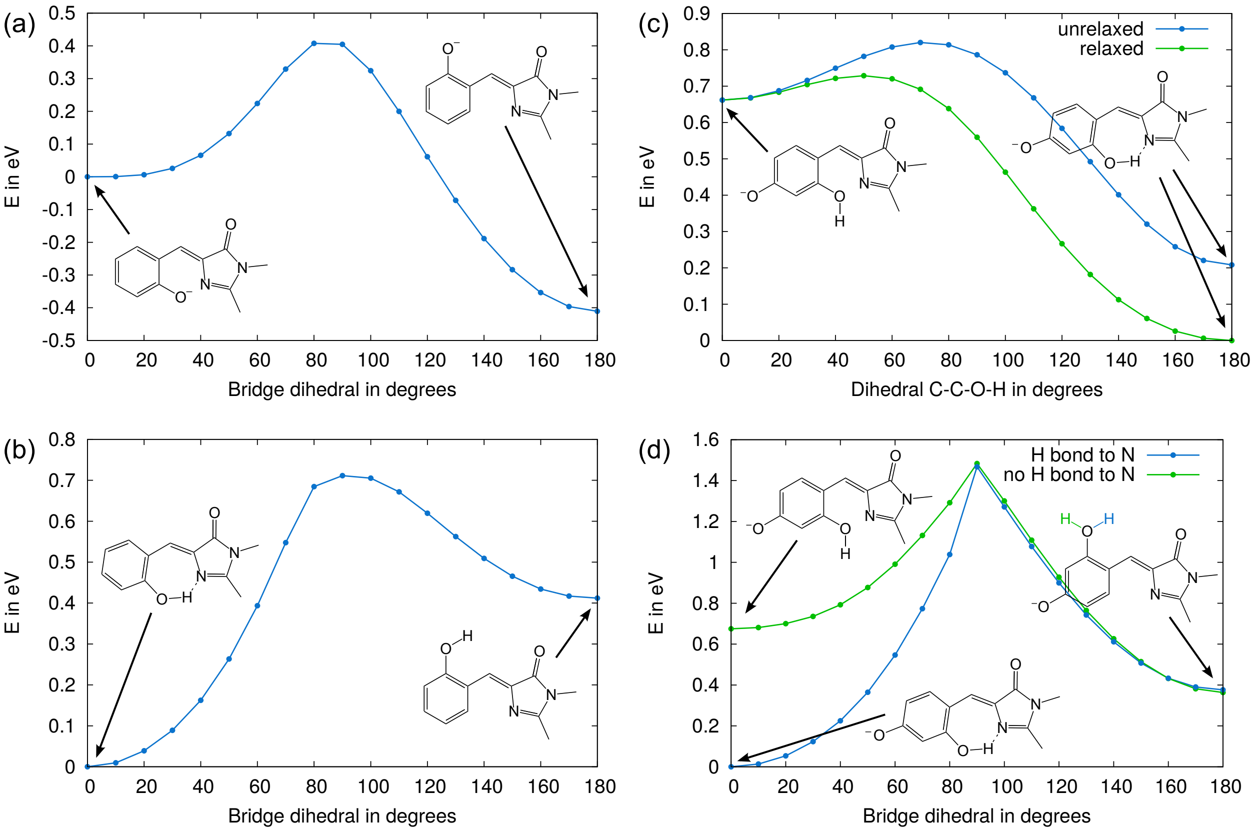

Fig. S1: Relaxed scans for the *cis*-*trans*-insomerisation along the bridge dihedral of atoms 2-3-4-5, cf. atom numbering in Fig. S2, in: (a) *o*-HBDI$^-$, (b) *o*-HBDI, and (d) *op*-DHBDI$^-$ with the two possible configurations of the *ortho*-OH group, i.e. H-bond to N or no H-bond. (c) Unrelaxed and relaxed scan around the CCOH dihedral angle of the *ortho*-OH group in *op*-DHBDI$^-$. The calculations of the scans in (b) and (d) were done with B3LYP/cc-pVDZ, the others with MP2/cc-pVDZ.

4 Geometrical data of the optimised structures

Atoms	*p*-HBDI⁻	*op*-DHBDI⁻	*cis o*-HBDI⁻
Angles (in degrees) between the atoms			
1-2-3	127.2	126.6	132.1
2-3-4	131.1	134.4	137.8
3-4-5	124.2	128.5	125.6
4-5-6	121.0	120.4	114.5
5-6-7	123.1	123.9	123.6
6-7-8	114.6	115.0	120.7
7-8-9	122.0	121.2	118.4
8-9-4	122.1	123.6	123.1
Distances (in Å) between the atoms			
1-2	1.4073	1.3997	1.4082
2-3	1.3979	1.4059	1.3928
3-4	1.4173	1.4099	1.4308
4-5	1.4383	1.4579	1.4780
5-6	1.3854	1.3877	1.4654
6-7	1.4630	1.4525	1.3885
7-8	1.4600	1.4658	1.4279
8-9	1.3857	1.3755	1.3906
9-4	1.4317	1.4504	1.4373
Selected distances in Å			
10-1	2.3714	2.6500	2.8861
11-1		1.6573	
C-O⁻	1.2688	1.2657	1.2622
C-O	1.2502	1.2518	1.2479

Table S1: Selected geometrical data of the optimised structures. Atoms are numbered as shown in the Fig. S2.

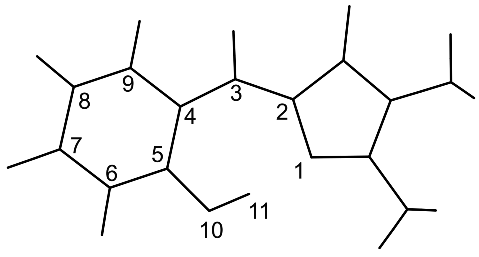

Fig. S2: Atom numbering used in Table S1 which is shown in the example of *op*-DHBDI⁻ and independent of the atom type and, thus, applicable to all geometries.

5 Ionisation potentials with state characters

	D_0			D_1			D_2			D_3		
	IP	S	C	IP	S	C	IP	S	C	IP	S	C
p-HBDI⁻	2.69	a''	π_4	4.51	a'	n_{O1}	4.87	a''	π_3	5.39	a''	π_2
cis o-HBDI⁻	2.54	a''	π_4	3.92	a'	n_{O1}	4.20	a''	π_3	5.33	a'	n_{O2}
trans o-HBDI⁻	2.62	a''	π_4	4.15	a'	n_{O1}	4.24	a''	π_3	5.45	a'	π_2
op-DHBDI⁻	2.90	a''	π_4	4.48	a''	π_3	4.64	a'	n_{O1}	5.16	a''	π_2

Table S2: Ionisation potentials of the HBDI⁻ analogues in eV, given with the symmetry of the states (S) and configurations (C). The configuration in each state corresponds to a hole in the given orbital. The corresponding orbitals are similar to those used in the ADC(2) calculations. These can be found in Figs. S3 to S6.

6 Excited states of *p*-HBDI⁻

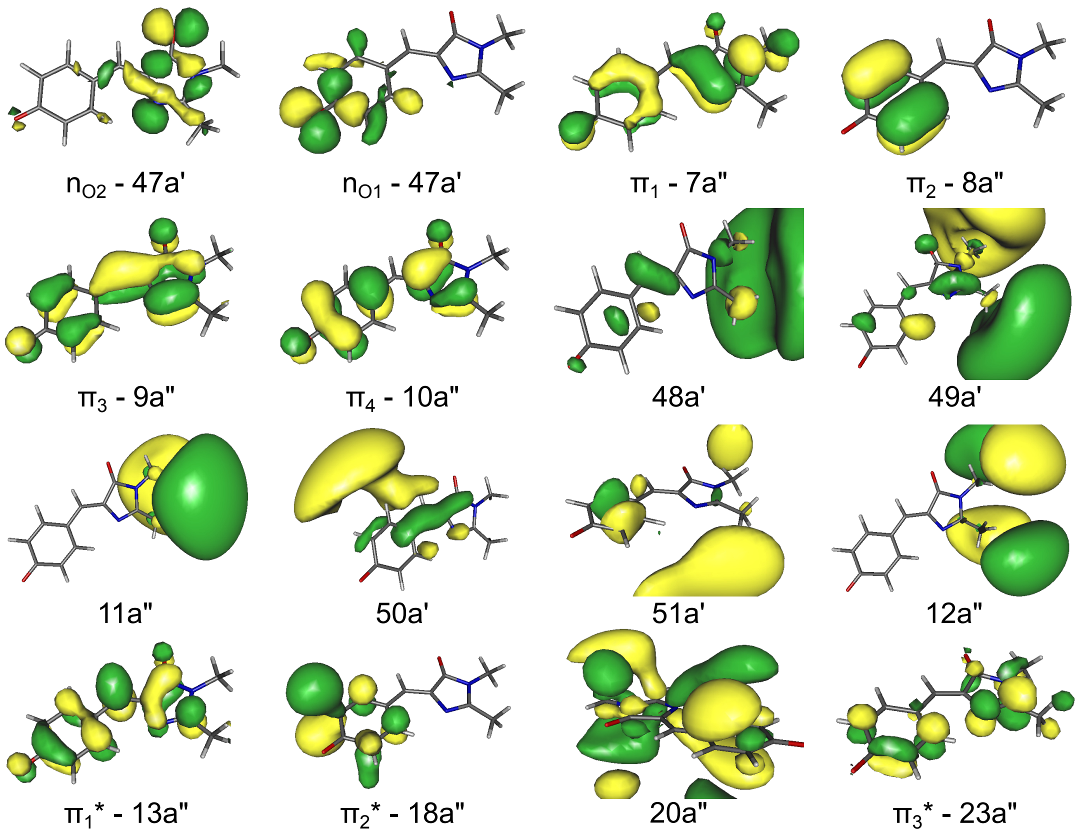

Fig. S3: HF orbitals used in the ADC(2)/aug-cc-pVDZ calculations for *p*-HBDI⁻

Table S3: Excited states of p-HBDI$^-$: Excitation energy (EE) in eV, symmetry (Sym), configuration with excitation (Exc) in terms of the orbitals in Fig. S3 with weight (c^2) in %, excitation; component (Comp; x,y, or z) and corresponding transition dipole moment (TDM) and transition strength; oscillator strength f.

EE	Sym	Exc	c^2	Exc	Comp	TDM	Trans. str.	f
2.53	$1a'$	$10a'' \to 13a''$	89	$\pi_4 \to \pi_1^*$	x	3.8933	15.1575	0.9483
					y	−0.3598	0.1295	
2.70	$1a''$	$10a'' \to 48a'$	84		z	0.0545	0.0030	0.0002
2.96	$2a''$	$47a' \to 13a''$	83	$n_{O1} \to \pi_1^*$	z	−0.0008	0.0000	0.0000
3.18	$3a''$	$10a'' \to 50a'$	78		z	−0.1054	0.0111	0.0009
3.22	$4a''$	$10a'' \to 49a'$	83		z	−0.0824	0.0068	0.0005
3.38	$2a'$	$10a'' \to 11a''$	91		x	0.1533	0.0235	
					y	0.0898	0.0081	0.0026
3.56	$5a''$	$10a'' \to 51a'$	65		z	0.0251	0.0006	0.0001
3.67	$6a''$	$10a'' \to 52a'$	46		z	0.1270	0.0161	0.0014
		$10a'' \to 53a'$	23					
3.69	$3a'$	$47a' \to 50a'$	52		x	−0.0021	0.0000	0.0014
		$47a' \to 48a'$	25		y	0.1256	0.0158	
3.77	$4a'$	$47a' \to 48a'$	56		x	−0.0418	0.0017	0.0003
		$47a' \to 50a'$	24		y	0.0419	0.0018	
3.91	$7a''$	$10a'' \to 52a'$	24		z	−0.0970	0.0094	0.0009
		$10a'' \to$ mix						
3.96	$5a'$	$47a' \to 51a'$	26		x	0.4474	0.2002	0.0230
		$10a'' \to 18a''$	15		y	−0.1924	0.0370	
3.99	$6a'$	$10a'' \to 18a''$	26	$\pi_4 \to \pi_2^*$	x	−0.4135	0.1709	0.0445
		$47a' \to 51a'$	13	$n_{O1} \to \sigma_{cont}^*$	y	0.5324	0.2835	
4.07	$8a''$	$10a'' \to 53a'$	50		z	−0.0360	0.0013	0.0001
		$10a'' \to 55a'$	26					
4.09	$7a'$	$10a'' \to 12a''$	78		x	−0.0087	0.0001	0.0185
					y	−0.4292	0.1842	
4.09	$9a''$	$46a' \to 13a''$	85		z	0.0653	0.0043	0.0004
4.24	$10a''$	$10a'' \to 54a'$	46		z	−0.0100	0.0001	0.0000
		$10a'' \to 55a'$	28					
4.25	$8a'$	$9a'' \to 13a''$	75	$\pi_3 \to \pi_1^*$	x	0.0214	0.0005	0.0001
					y	−0.0232	0.0005	
4.27	$11a''$	$47a' \to 18a''$	48		z	−0.0750	0.0056	0.0006
		$47a' \to 15a''$	14					
4.28	$9a'$	$47a' \to 49a'$	25		x	0.0650	0.0042	0.0079
		$47a' \to 52a'$	25		y	−0.2660	0.0708	
4.33	$10a'$	$47a' \to 49a'$	41		x	0.0636	0.0040	0.0005
		$47a' \to 53a'$	34		y	−0.0193	0.0004	
4.38	$12a''$	$9a'' \to 48a'$	54		z	−0.1616	0.0261	0.0028
4.40	$13a''$	$47a' \to 11a''$	93		z	−0.0031	0.0000	0.0000
4.45	$11a'$	$10a'' \to 14a''$	39		x	0.1892	0.0358	0.0074
		$46a' \to 48a'$	32		y	0.1786	0.0319	
4.45	$12a'$	$46a' \to 48a'$	45		x	0.0862	0.0074	0.0061
		$10a'' \to 14a''$	29		y	0.2194	0.0481	
4.48	$14a''$	$10a'' \to 57a'$	46		z	0.0372	0.0014	0.0002
		$9a'' \to 48a'$	16					
4.74	$13a'$	$8a'' \to 13a''$	81	$\pi_2 \to \pi_1^*$	x	0.3143	0.0988	0.0229
					y	−0.3137	0.0984	
4.74	$15a''$	$10a'' \to 56a'$	74		z	−0.1160	0.0134	0.0016

Table S3: Excited states of *p*-HBDI⁻ continued

EE	Sym	Exc	c^2	Exc	Comp	TDM	Trans. str.	f
4.75	$14a'$	$47a' \to 55a'$	48		x	0.0692	0.0048	0.0020
		$47a' \to 54a'$	17		y	−0.1114	0.0124	
4.82	$15a'$	$47a' \to 51a'$	26		x	−0.0209	0.0004	0.0009
		$47a' \to$ mix			y	−0.0869	0.0076	
4.95	$16a'$	$10a'' \to 23a''$	30	$\pi_4 \to \pi_3^*$	x	0.3657	0.1338	0.0735
		$10a'' \to 20a''$	18	$\pi_4 \to \pi_{\text{cont}}^*$	y	0.6873	0.4724	
4.98	$16a''$	$9a'' \to 49a'$	69		z	−0.0398	0.0016	0.0002
4.98	$17a'$	$47a' \to 52a'$	28		x	0.1104	0.0122	0.0016
		$47a' \to 56a'$	22		y	−0.0295	0.0009	
5.00	$18a'$	$46a' \to 49a'$	69	$n_{\text{O2}} \to \sigma_{\text{cont}}^*$	x	0.2099	0.0440	0.0114
					y	0.2220	0.0493	
5.01	$17a''$	$47a' \to 12a''$	38		z	−0.0007	0.0000	0.0000
		$47a' \to$ mix						
5.01	$18a''$	$10a'' \to 58a'$	31		z	−0.0251	0.0006	0.0001
		$10a'' \to 57a'$	22					
5.01	$19a'$	$10a'' \to 15a''$	39		x	−0.0558	0.0031	0.0005
		$10a'' \to 14a''$	15		y	−0.0286	0.0008	
5.06	$19a''$	$47a' \to 12a''$	53		z	0.0164	0.0003	0.0000
		$47a' \to 15a''$	11					
5.09	$20a'$	$9a'' \to 11a''$	72		x	0.0928	0.0086	0.0015
					y	−0.0561	0.0031	
5.14	$20a''$	$9a'' \to 50a'$	53		z	0.0559	0.0031	0.0004
5.15	$21a''$	$46a' \to 11a''$	85		z	−0.0300	0.0009	0.0001
5.21	$22a''$	$10a'' \to 60a'$	38		z	−0.1581	0.0250	0.0032
		$10a'' \to 61a'$	23					
5.21	$21a'$	$47a' \to 57a'$	25		x	−0.0053	0.0000	0.0018
		$47a' \to$ mix			y	0.1192	0.0142	
5.25	$22a'$	$46a' \to 50a'$	33		x	−0.0778	0.0061	0.0067
		$46a' \to$ mix			y	0.2152	0.0463	
5.35	$23a''$	$10a'' \to 59a'$	38		z	0.1091	0.0119	0.0016
		$10a'' \to 58a'$	22					
5.38	$23a'$	$7a'' \to 13a''$	75		x	−0.4079	0.1664	0.0900
					y	0.7183	0.5159	
5.39	$24a''$	$10a'' \to 61a'$	46		z	0.1226	0.0150	0.0020
		$10a'' \to 60a'$	11					
5.44	$25a''$	$9a'' \to 51a'$	37		z	0.0052	0.0000	0.0000
		$8a'' \to 50a'$	21					
5.52	$26a''$	$8a'' \to 48a'$	39		z	−0.0200	0.0004	0.0001
5.52	$24a'$	$10a'' \to 16a''$	53	$\pi_4 \to \pi_{\text{cont}}^*$	x	−0.2505	0.0628	0.0104
		$10a'' \to 19a''$	16		y	−0.1173	0.0138	
5.54	$25a'$	$47a' \to 56a'$	39		x	0.1102	0.0121	0.0017
		$47a' \to 54a'$	23		y	−0.0127	0.0002	
5.55	$27a''$	$9a'' \to 51a'$	35		z	0.1616	0.0261	0.0036
5.59	$28a''$	$45a' \to 13a''$	47		z	0.0800	0.0064	0.0009
		$44a' \to 13a''$	16					

7 Excited states of *cis* *o*-HBDI^{-}

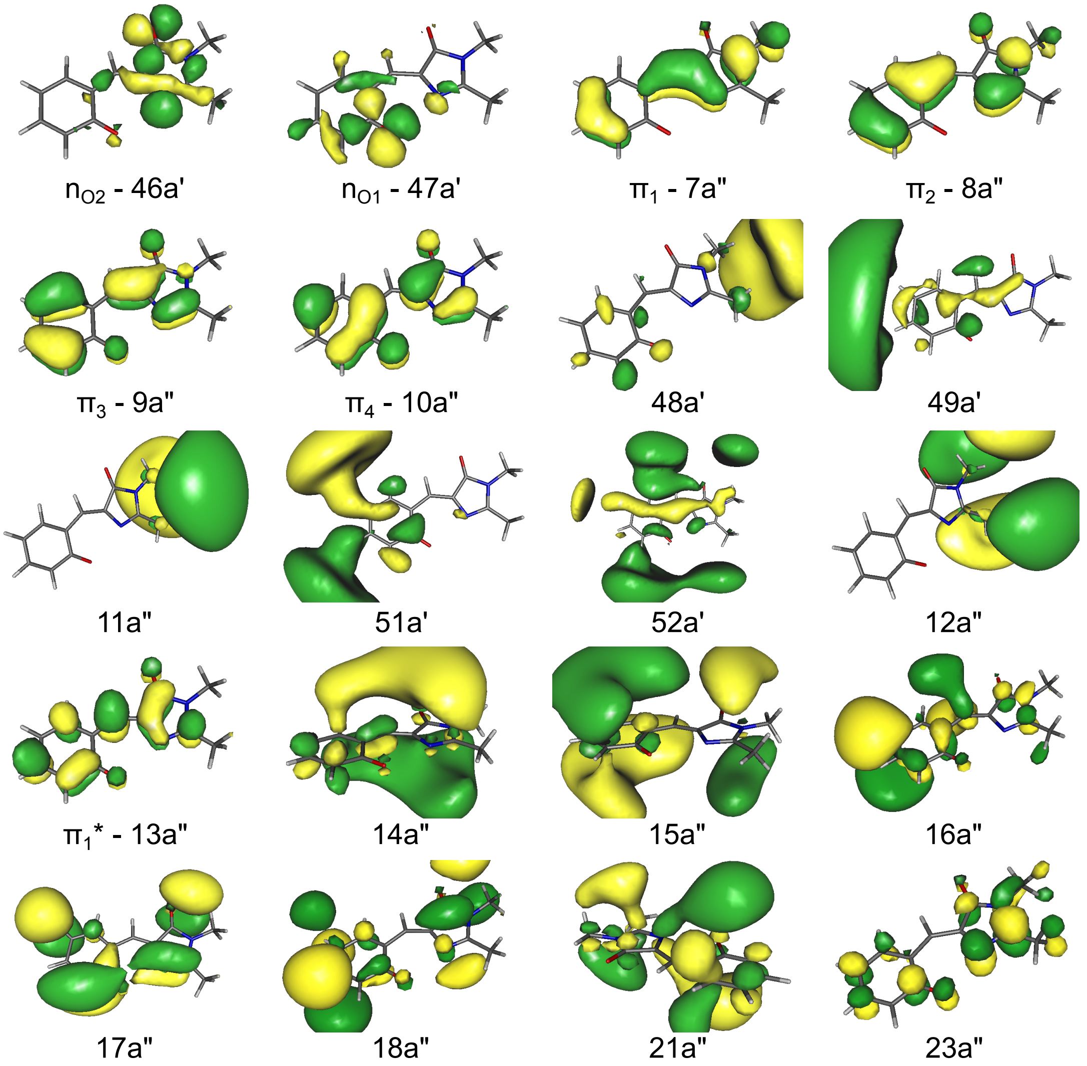

Fig. S4: HF orbitals used in the ADC(2)/aug-cc-pVDZ calculations for *cis* *o*-HBDI^{-}

Table S4: Excited states of *cis* *o*-HBDI^{-}

EE	Sym	Exc	c^2	Exc	Comp	TDM	Trans. str.	*f*
2.36	$1a'$	$10a'' \rightarrow 13a''$	87	$\pi_4 \rightarrow \pi_1^*$	x	−2.7652	7.6465	0.4429
					y	−0.0844	0.0071	
2.44	$1a''$	$47a' \rightarrow 13a''$	89	$n_{O1} \rightarrow \pi_1^*$	z	0.0151	0.0002	0.0000
2.76	$2a''$	$10a'' \rightarrow 48a'$	72		z	−0.0836	0.0070	0.0005
2.79	$3a''$	$10a'' \rightarrow 49a'$	67		z	0.1408	0.0198	0.0014
		$10a'' \rightarrow 48a'$	15					
3.07	$2a'$	$47a' \rightarrow 49a'$	78		x	−0.0571	0.0033	0.0006
					y	0.0707	0.0050	
3.18	$3a'$	$47a' \rightarrow 48a'$	89		x	−0.0075	0.0001	0.0001
					y	−0.0306	0.0009	

Table S4: Excited states of *cis o*-HBDI⁻ continued

EE	Sym	Exc	c^2	Exc	Comp	TDM	Trans. str.	f
3.18	$4a''$	$10a'' \to 51a'$	65		z	−0.1178	0.0139	0.0011
		$10a'' \to 50a'$	18					
3.33	$5a''$	$10a'' \to 50a'$	68		z	0.1490	0.0222	0.0018
		$10a'' \to 51a'$	17					
3.41	$4a'$	$10a'' \to 11a''$	91		x	−0.0024	0.0000	0.0012
					y	−0.1208	0.0146	
3.43	$5a'$	$47a' \to 51a'$	71		x	0.1421	0.0202	0.0065
					y	0.2389	0.0571	
3.46	$6a''$	$10a'' \to 52a'$	67		z	−0.1613	0.0260	0.0022
3.66	$6a'$	$47a' \to 50a'$	35		x	0.1714	0.0294	0.0029
		$47a' \to 52a'$	26		y	0.0537	0.0029	
3.72	$7a'$	$47a' \to 52a'$	40		x	0.1566	0.0245	0.0023
		$47a' \to 50a'$	39		y	−0.0134	0.0002	
3.80	$7a''$	$47a' \to 11a''$	90		z	−0.0185	0.0003	0.0000
3.81	$8a''$	$10a'' \to 54a'$	35		z	−0.0065	0.0000	0.0000
		$10a'' \to 53a'$	27					
3.88	$8a'$	$9a'' \to 13a''$	65	$\pi_3 \to \pi_1^*$	x	1.2750	1.6257	0.1743
					y	−0.4550	0.2070	
3.92	$9a''$	$10a'' \to 54a'$	43		z	−0.0400	0.0016	0.0002
		$10a'' \to 53a'$	30					
3.94	$10a''$	$46a' \to 13a''$	77	$n_{O2} \to \pi_1^*$	z	−0.0488	0.0024	0.0002
4.04	$11a''$	$10a'' \to 55a'$	29		z	0.0233	0.0005	0.0001
		$10a'' \to 53a'$	28					
4.08	$9a'$	$47a' \to 54a'$	60		x	−0.1054	0.0111	0.0035
					y	0.1536	0.0236	
4.10	$10a'$	$10a'' \to 12a''$	75		x	−0.1513	0.0229	0.0074
					y	−0.2261	0.0511	
4.11	$12a''$	$47a' \to 17a''$	15		z	−0.0738	0.0054	0.0005
		$47a' \to$ mix						
4.14	$13a''$	$9a'' \to 48a'$	58		z	−0.1010	0.0102	0.0010
		$8a'' \to 48a'$	15					
4.16	$11a'$	$10a'' \to 14a''$	21	$\pi_4 \to \pi_{cont}^*$	x	0.5009	0.2509	0.0261
		$10a'' \to 15a''/12a''$	15/14		y	−0.0768	0.0059	
4.24	$12a'$	$47a' \to 53a'$	54		x	0.0180	0.0003	0.0007
		$47a' \to 56a'$	19		y	−0.0825	0.0068	
4.34	$13a'$	$46a' \to 48a'$	82		x	−0.0869	0.0076	0.0012
					y	0.0612	0.0037	
4.36	$14a''$	$10a'' \to 58a'$	45		z	0.0189	0.0004	0.0000
		$10a'' \to$ mix						
4.41	$14a'$	$47a' \to 55a'$	44		x	−0.1565	0.0245	0.0028
		$47a' \to$ mix			y	−0.0320	0.0010	
4.44	$15a''$	$9a'' \to 49a'$	68		z	−0.0294	0.0009	0.0001
4.45	$16a''$	$47a' \to 12a''$	90		z	−0.0093	0.0001	0.0000
4.46	$17a''$	$10a'' \to 56a'$	42		z	0.0231	0.0005	0.0001
		$10a'' \to 55a'$	39					
4.67	$15a'$	$10a'' \to 14a''$	22	$\pi_4 \to \pi_{cont}^*$	x	−0.0716	0.0051	0.0206
		$10a'' \to 16a''$	13		y	−0.4182	0.1749	
4.67	$16a'$	$47a' \to 58a'$	40		x	−0.0165	0.0003	0.0051
		$47a' \to$ mix			y	0.2112	0.0446	
4.71	$18a''$	$9a'' \to 50a'$	59		z	−0.1920	0.0369	0.0043

Table S4: Excited states of *cis o*-HBDI⁻ continued

EE	Sym	Exc	c^2	Exc	Comp	TDM	Trans. str.	f
4.77	$17a'$	$47a' \to 56a'$	42		x	−0.0742	0.0055	0.0007
		$47a' \to 55a'$	29		y	0.0194	0.0004	
4.78	$18a'$	$8a'' \to 13a''$	25		x	0.2901	0.0842	0.0099
					y	−0.0110	0.0001	
4.79	$19a''$	$10a'' \to 57a'$	29		z	0.0661	0.0044	0.0005
		$9a'' \to 51a'$	21					
4.81	$20a''$	$47a' \to 18a''$	16		z	−0.0086	0.0001	0.0000
		$47a' \to$ mix						
4.81	$19a'$	$9a'' \to 11a''$	69		x	0.1864	0.0348	0.0042
		$8a'' \to 11a''$	16		y	−0.0310	0.0010	
4.85	$21a''$	$9a'' \to 51a'$	34		z	−0.1371	0.0188	0.0022
		$10a'' \to 57a'$	32					
4.89	$20a'$	$46a' \to 50a'$	68		x	−0.0353	0.0012	0.0111
					y	−0.3028	0.0917	
4.93	$21a'$	$10a'' \to 15a''$	40	$\pi_4 \to \pi^*_{\mathrm{cont}}$	x	−1.0743	1.1541	0.2487
		$10a'' \to 14a''$	22		y	0.9514	0.9052	
5.00	$22a'$	$8a'' \to 13a''$	19	$\pi_2 \to \pi^*_1$	x	−1.4229	2.0248	0.4115
		$10a'' \to 18a''/15a''$	16/11	$\pi_4 \to \pi^*$	y	1.1558	1.3358	
5.02	$22a''$	$46a' \to 11a''$	88		z	0.0242	0.0006	0.0001
5.04	$23a''$	$9a'' \to 52a'$	38		z	−0.1535	0.0235	0.0029
		$9a'' \to 51a'$	15					
5.15	$23a'$	$46a' \to 52a'$	24	$n_{\mathrm{O2}} \to \sigma^*$	x	0.2836	0.0804	0.0243
		$46a' \to$ mix			y	−0.3357	0.1127	
5.19	$24a'$	$47a' \to 57a'$	72		x	0.0833	0.0069	0.0021
					y	−0.0985	0.0097	
5.20	$24a''$	$8a'' \to 48a'$	37		z	−0.1742	0.0304	0.0039
		$9a'' \to 48a'$	23					
5.24	$25a''$	$10a'' \to 59a'$	31		z	0.0055	0.0000	0.0000
		$10a'' \to 58a'$	13					
5.27	$26a''$	$10a'' \to 60a'$	27		z	−0.0825	0.0068	0.0009
		$10a'' \to 62a'$	24					
5.28	$25a'$	$10a'' \to 23a''$	21	$\pi_4 \to \pi^*$	x	−0.0256	0.0007	0.0341
		$10a'' \to 17a''/21a''/16a''$	12/12/11	$\pi_4 \to \pi^*_{\mathrm{cont}}$	y	−0.5130	0.2632	
5.30	$27a''$	$45a' \to 13a''$	64		z	0.0760	0.0058	0.0008
5.35	$28a''$	$10a'' \to 62a'$	23		z	0.0401	0.0016	0.0002
		$10a'' \to 65a'$	21					
5.38	$29a''$	$9a'' \to 53a'$	20		z	−0.0101	0.0001	0.0000
		$9a'' \to 52a'$	15					
5.43	$26a'$	$46a' \to 49a'$	60		x	−0.0568	0.0032	0.0013
					y	0.0797	0.0064	
5.51	$27a'$	$47a' \to 59a'$	29		x	−0.0558	0.0031	0.0019
		$47a' \to 61a'$	22		y	−0.1049	0.0110	
5.54	$28a'$	$9a'' \to 12a''$	72		x	−0.2216	0.0491	0.0067
		$8a'' \to 12a''$	13		y	−0.0185	0.0003	
5.57	$29a'$	$10a'' \to 16a''$	19		x	0.1306	0.0171	0.0053
		$10a'' \to 20a''/17a''/21a''$	15/13/13		y	−0.1465	0.0215	
5.61	$30a'$	$46a' \to 53a'$	17		x	0.1052	0.0111	0.0023
		$47a' \to$ mix			y	−0.0736	0.0054	

7.1 Excited states of *trans o*-HBDI⁻

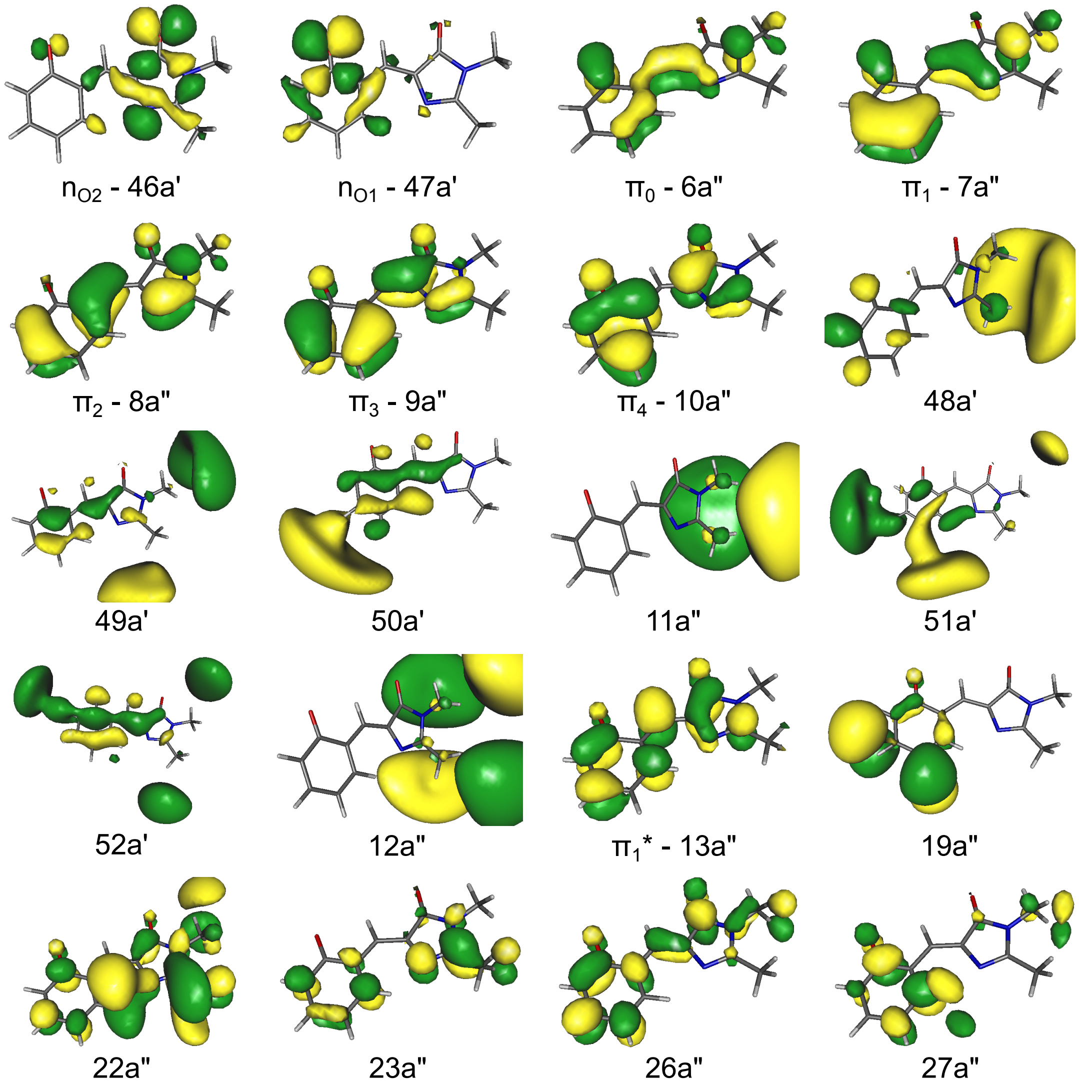

Fig. S5: HF orbitals used in the ADC(2)/aug-cc-pVDZ calculations for *trans o*-HBDI$^-$

Table S5: Excited states of *trans o*-HBDI$^-$

EE	Sym	Exc	c^2	Exc	Comp	TDM	Trans. str.	*f*
2.09	$1a'$	$10a''\rightarrow 13a''$	88	$\pi_4\rightarrow\pi_1^*$	x	−1.6431	2.6998	0.3986
					y	2.2560	5.0895	
2.37	$1a''$	$47a'\rightarrow 13a''$	91	$n_{O1}\rightarrow\pi_1^*$	z	−0.0252	0.0006	0.0000
2.63	$2a''$	$10a''\rightarrow 48a'$	83		z	−0.0909	0.0083	0.0005
2.85	$3a''$	$10a''\rightarrow 50a'$	48		z	−0.1196	0.0143	0.0010
		$10a''\rightarrow 49a'$	35					
3.14	$4a''$	$10a''\rightarrow 49a'$	46		z	−0.1033	0.0107	0.0008
		$10a''\rightarrow 50a'$	28					
3.29	$2a'$	$47a'\rightarrow 50a'$	40		x	0.0869	0.0076	0.0006
		$47a'\rightarrow 48a'$	29		y	0.0150	0.0002	
3.32	$3a'$	$10a''\rightarrow 11a''$	91		x	0.0654	0.0043	0.0003
					y	−0.0025	0.0000	

Table S5: Excited states of *trans* *o*-HBDI⁻ continued

EE	Sym	Exc	c^2	Exc	Comp	TDM	Trans. str.	f
3.37	$4a'$	$47a' \rightarrow 48a'$	60		x	−0.0896	0.0080	0.0007
		$47a' \rightarrow 50a'$	22		y	−0.0147	0.0002	
3.40	$5a''$	$10a'' \rightarrow 51a'$	64		z	−0.1148	0.0132	0.0011
3.49	$6a''$	$10a'' \rightarrow 54a'$	37		z	−0.0377	0.0014	0.0001
		$10a'' \rightarrow 52a'$	22					
3.67	$5a'$	$9a'' \rightarrow 13a''$	63	$\pi_3 \rightarrow \pi_1^*$	x	0.4644	0.2156	0.2116
					y	−1.4629	2.1400	
3.68	$6a'$	$47a' \rightarrow 49a'$	31	$n_{O1} \rightarrow \sigma^*$	x	0.0430	0.0018	0.0433
		$47a' \rightarrow 51a'$	25		y	−0.6917	0.4785	
3.78	$7a''$	$10a'' \rightarrow 52a'$	34		z	−0.0795	0.0063	0.0006
		$10a'' \rightarrow 54a'$	24					
3.90	$7a'$	$47a' \rightarrow 52a'$	24		x	−0.1629	0.0266	0.0030
		$47a' \rightarrow 50a'/53a'$	23/21		y	0.0667	0.0044	
3.93	$8a''$	$46a' \rightarrow 13a''$	83		z	−0.0636	0.0041	0.0004
3.95	$9a''$	$10a'' \rightarrow 53a'$	50		z	−0.1259	0.0158	0.0015
		$10a'' \rightarrow 52a'$	27					
4.00	$8a'$	$10a'' \rightarrow 12a''$	84		x	−0.0782	0.0061	0.0028
					y	−0.1505	0.0226	
4.00	$10a''$	$47a' \rightarrow 11a''$	94		z	−0.0107	0.0001	0.0000
4.00	$9a'$	$47a' \rightarrow 51a'$	35		x	−0.0191	0.0004	0.0023
		$47a' \rightarrow 54a'$	22		y	−0.1520	0.0231	
4.03	$11a''$	$9a'' \rightarrow 48a'$	61		z	−0.0867	0.0075	0.0007
4.12	$12a''$	$10a'' \rightarrow 55a'/56a'$	44/30		z	0.0757	0.0057	0.0006
4.26	$10a'$	$10a'' \rightarrow 14a''$	22	$\pi_4 \rightarrow \pi_{cont}^*$	x	0.2954	0.0873	0.0156
					y	0.2495	0.0623	
4.30	$13a''$	$10a'' \rightarrow 57a'$	51		z	−0.0088	0.0001	0.0000
4.30	$11a'$	$47a' \rightarrow 54a'$	27		x	−0.0148	0.0002	0.0004
		$47a' \rightarrow 53a'/55a'$	13/11		y	0.0574	0.0033	
4.40	$12a'$	$46a' \rightarrow 48a'$	79		x	−0.0940	0.0088	0.0010
					y	0.0254	0.0006	
4.43	$14a''$	$9a'' \rightarrow 49a'$	50		z	0.0709	0.0050	0.0005
		$9a'' \rightarrow 50a'$	29					
4.48	$15a''$	$47a' \rightarrow 18a''$	22		z	−0.0433	0.0019	0.0002
		$47a' \rightarrow 27a''/21a''$	14/10					
4.54	$13a'$	$47a' \rightarrow 52a'$	32		x	−0.1093	0.0119	0.0016
		$47a' \rightarrow 55a'$	17		y	0.0480	0.0023	
4.54	$14a'$	$8a'' \rightarrow 13a''$	36	$\pi_2 \rightarrow \pi_1^*$	x	−0.3616	0.1308	0.0170
		$47a' \rightarrow 52a'$	15	$n_{O2} \rightarrow \sigma^*$	y	0.1471	0.0216	
4.56	$16a''$	$10a'' \rightarrow 56a'$	48		z	0.0188	0.0004	0.0000
		$10a'' \rightarrow 55a'$	21					
4.59	$15a'$	$47a' \rightarrow 56a'$	32		x	−0.1596	0.0255	0.0151
		$47a' \rightarrow 55a'/53a'$	23/16		y	0.3301	0.1090	
4.62	$17a''$	$9a'' \rightarrow 50a'$	51		z	−0.0606	0.0037	0.0004
		$9a'' \rightarrow 49a'$	22					
4.65	$18a''$	$47a' \rightarrow 12a''$	89		z	0.0117	0.0001	0.0000
4.71	$16a'$	$10a'' \rightarrow 14a''$	35		x	0.1408	0.0198	0.0023
		$10a'' \rightarrow 20a''/15a''$	13/11		y	0.0136	0.0002	
4.72	$17a'$	$9a'' \rightarrow 11a''$	65	$\pi_3 \rightarrow \pi_{cont}^*$	x	−0.0477	0.0023	0.0142
		$8a'' \rightarrow 11a''$	14	$\pi_2 \rightarrow \pi_{cont}^*$	y	−0.3467	0.1202	

Table S5: Excited states of *trans o*-HBDI⁻ continued

EE	Sym	Exc	c^2	Exc	Comp	TDM	Trans. str.	f
4.76	$18a'$	$10a''\rightarrow22a''$	18	$\pi_4\rightarrow\pi^*$	x	−0.3006	0.0903	0.0569
		$8a''\rightarrow13a''$	10	$\pi_2\rightarrow\pi_1^*$	y	−0.6306	0.3977	
4.89	$19a'$	$47a'\rightarrow57a'$	46		x	0.0696	0.0048	0.0006
		$47a'\rightarrow54a'$	13		y	−0.0044	0.0000	
4.91	$19a''$	$9a''\rightarrow51a'$	58	$\pi_3\rightarrow\sigma^*$	z	−0.2940	0.0864	0.0104
4.96	$20a'$	$46a'\rightarrow49a'$	68		x	−0.0519	0.0027	0.0040
					y	−0.1748	0.0305	
4.97	$20a''$	$47a'\rightarrow19a''$	25		z	0.0076	0.0001	0.0000
		$47a'\rightarrow26a''/14a''$	16/14					
4.99	$21a'$	$10a''\rightarrow15a''$	55		x	0.6792	0.4614	0.0960
		$10a''\rightarrow14a''$	15		y	0.5696	0.3244	
4.99	$21a''$	$10a''\rightarrow60a'/58a'$	21/21		z	0.0109	0.0001	0.0000
5.02	$22a''$	$9a''\rightarrow54a'$	28		z	0.0368	0.0014	0.0002
5.05	$23a''$	$8a''\rightarrow48a'$	45		z	−0.1794	0.0322	0.0040
		$9a''\rightarrow48a'$	13					
5.10	$22a'$	$47a'\rightarrow55a'$	33		x	−0.1348	0.0182	0.0064
		$47a'\rightarrow56a'$	28		y	−0.1821	0.0331	
5.10	$24a''$	$46a'\rightarrow11a''$	90		z	0.0349	0.0012	0.0002
5.12	$23a'$	$10a''\rightarrow19a''$	21	$\pi_4\rightarrow\pi^*_{cont}$	x	−0.6647	0.4418	0.1513
		$10a''\rightarrow26a''$	20	$\pi_4\rightarrow\pi^*$	y	−0.8749	0.7654	
5.12	$25a''$	$10a''\rightarrow59a'$	39		z	0.0788	0.0062	0.0008
		$10a''\rightarrow$mix						
5.25	$26a''$	$47a'\rightarrow15a''/14a''$	33/25		z	−0.0236	0.0006	0.0001
5.28	$27a''$	$10a''\rightarrow61a'/62a'$	24/19		z	−0.0041	0.0000	0.0000
5.30	$28a''$	$9a''\rightarrow52a'$	31		z	−0.0262	0.0007	0.0001
5.31	$29a''$	$9a''\rightarrow52a'$	28		z	−0.0541	0.0029	0.0004
		$10a''\rightarrow61a'$	18					
5.33	$24a'$	$46a'\rightarrow54a'$	36		x	−0.2021	0.0408	0.0075
		$46a'\rightarrow$mix			y	−0.1286	0.0165	
5.42	$25a'$	$9a''\rightarrow12a''$	73		x	0.1117	0.0125	0.0043
					y	−0.1397	0.0195	
5.48	$30a''$	$9a''\rightarrow53a'$	61		z	−0.0359	0.0013	0.0002
5.53	$26a'$	$46a'\rightarrow50a'$	22		x	0.0593	0.0035	0.0008
		$46a'\rightarrow$mix			y	−0.0509	0.0026	
5.55	$27a'$	$47a'\rightarrow58a'$	24		x	0.1282	0.0164	0.0033
		$47a'\rightarrow$mix			y	0.0865	0.0075	
5.59	$28a'$	$6a''\rightarrow13a''$	32	$\pi_0\rightarrow\pi_1^*$	x	−0.2692	0.0724	0.0631
		$7a''\rightarrow13a''$	19	$\pi_1\rightarrow\pi_1^*$	y	0.6234	0.3886	
5.65	$29a'$	$47a'\rightarrow59a'$	35		x	−0.0374	0.0014	0.0018
		$47a'\rightarrow61a'$	28		y	−0.1073	0.0115	
5.68	$30a'$	$10a''\rightarrow16a''$	23		x	0.1556	0.0242	0.0093

8 Excited states of *op*-DHBDI⁻

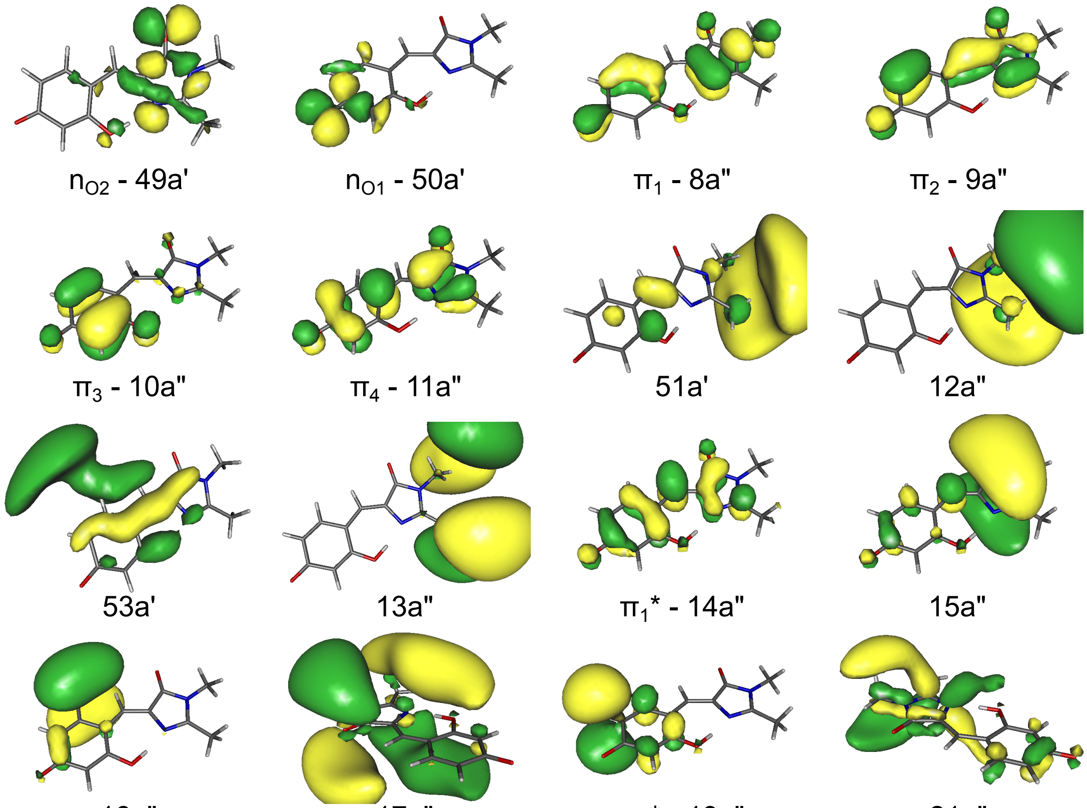

Fig. S6: HF orbitals used in the ADC(2)/aug-cc-pVDZ calculations for *op*-DHBDI⁻

Table S6: Excited states of *op*-DHBDI⁻

EE	Sym	Exc	c^2	Exc	Comp	TDM	Trans. str.	*f*
2.65	$1a'$	$11a'' \rightarrow 14a''$	83	$\pi_4 \rightarrow \pi_1^*$	x	3.8822	15.0712	0.9848
					y	−0.2634	0.0694	
2.78	$1a''$	$11a'' \rightarrow 51a'$	84		z	0.0479	0.0023	0.0002
2.99	$2a''$	$50a' \rightarrow 14a''$	77	$n_{O1} \rightarrow \pi_1^*$	z	0.0032	0.0000	0.0000
3.33	$3a''$	$11a'' \rightarrow 53a'$	49		z	0.0173	0.0003	0.0000
		$11a'' \rightarrow 52a'$	29					
3.40	$4a''$	$11a'' \rightarrow 52a'$	60		z	−0.1162	0.0135	0.0011
		$11a'' \rightarrow 53a'$	25					
3.46	$2a'$	$11a'' \rightarrow 12a''$	90		x	0.0747	0.0056	0.0013
					y	0.1014	0.0103	
3.63	$3a'$	$10a'' \rightarrow 14a''$	81	$\pi_3 \rightarrow \pi_1^*$	x	0.0942	0.0089	0.0029
					y	−0.1535	0.0235	
3.76	$5a''$	$11a'' \rightarrow 56a'$	27		z	−0.0922	0.0085	0.0008
		$11a'' \rightarrow 54a'$	26					
3.82	$4a'$	$50a' \rightarrow 51a'$	60		x	0.0652	0.0042	0.0012
		$50a' \rightarrow 53a'$	28		y	−0.0929	0.0086	

Table S6: Excited states of *op*-DHBDI$^-$ continued

EE	Sym	Exc	c^2	Exc	Comp	TDM	Trans. str.	f
3.86	$5a'$	$50a' \rightarrow 53a'$	53		x	−0.0883	0.0078	0.0022
		$50a' \rightarrow 51a'$	29		y	0.1260	0.0159	
3.89	$6a''$	$11a'' \rightarrow 55a'$	61		z	0.0977	0.0095	0.0009
		$11a'' \rightarrow 54a'$	18					
4.04	$7a''$	$11a'' \rightarrow 54a'$	46		z	−0.0917	0.0084	0.0008
		$11a'' \rightarrow 57a'/55a'$	16/13					
4.15	$6a'$	$11a'' \rightarrow 13a''$	79	$\pi_4 \rightarrow \pi^*_{\text{cont}}$	x	−0.2691	0.0724	0.0176
					y	−0.3175	0.1008	
4.18	$7a'$	$50a' \rightarrow 55a'$	36		x	0.0583	0.0034	0.0011
		$50a' \rightarrow 52a'$	22		y	0.0830	0.0069	
4.22	$8a''$	$10a'' \rightarrow 51a'$	42		z	−0.0578	0.0033	0.0003
		$11a'' \rightarrow 56a'$	15					
4.27	$9a''$	$11a'' \rightarrow 56a'$	25		z	−0.0022	0.0000	0.0000
		mix						
4.28	$10a''$	$49a' \rightarrow 14a''$	78		z	0.0540	0.0029	0.0003
4.31	$8a'$	$11a'' \rightarrow 19a''$	25	$\pi_4 \rightarrow \pi^*_2$	x	−0.4043	0.1634	0.0390
		$11a'' \rightarrow 16a''/15a''$	19/15	$\pi_4 \rightarrow \pi^*_{\text{cont}}$	y	0.4537	0.2058	
4.39	$9a'$	$50a' \rightarrow 52a'$	56		x	0.0153	0.0002	0.0046
		$50a' \rightarrow 55a'$	17		y	−0.2052	0.0421	
4.44	$11a''$	$11a'' \rightarrow 57a'$	41		z	0.0205	0.0004	0.0000
		$11a'' \rightarrow 58a'$	34					
4.45	$10a'$	$50a' \rightarrow 56a'$	56		x	0.0505	0.0025	0.0003
		$50a' \rightarrow 54a'$	14		y	0.0197	0.0004	
4.46	$12a''$	$50a' \rightarrow 12a''$	93		z	−0.0010	0.0000	0.0000
4.49	$11a'$	$9a'' \rightarrow 14a''$	69		x	−0.0829	0.0069	0.0037
					y	−0.1622	0.0263	
4.56	$13a''$	$50a' \rightarrow 19a''$	36		z	−0.0643	0.0041	0.0005
		$50a' \rightarrow 16a''$	20					
4.58	$12a'$	$49a' \rightarrow 51a'$	74		x	0.1129	0.0127	0.0023
					y	0.0893	0.0080	
4.60	$14a''$	$11a'' \rightarrow 60a'$	44		z	0.0735	0.0054	0.0006
		$11a'' \rightarrow 63a'$	13					
4.66	$13a'$	$11a'' \rightarrow 15a''$	36	$\pi_4 \rightarrow \pi^*_{\text{cont}}$	x	0.2567	0.0659	0.0666
		$11a'' \rightarrow 24a''/19a''$	14/11	$\pi_4 \rightarrow \pi^*_3/\pi^*_2$	y	0.7196	0.5179	
4.67	$15a''$	$10a'' \rightarrow 53a'$	40		z	−0.1231	0.0151	0.0017
		$9a'' \rightarrow 51a'$	14					
4.72	$16a''$	$10a'' \rightarrow 53a'$	30		z	0.0402	0.0016	0.0002
		$10a'' \rightarrow 52a'$	26					
4.78	$14a'$	$11a'' \rightarrow 21a''$	26	$\pi_4 \rightarrow \pi^*_{\text{cont}}$	x	−0.1485	0.0221	0.0236
		$11a'' \rightarrow 15a''$	22		y	−0.4232	0.1791	
4.83	$17a''$	$9a'' \rightarrow 51a'$	28		z	−0.1010	0.0102	0.0012
		$10a'' \rightarrow 52a'/51a'$	23/16					
4.86	$15a'$	$50a' \rightarrow 58a'$	40		x	0.0772	0.0060	0.0011
		$50a' \rightarrow 57a'$	32		y	−0.0551	0.0030	
4.90	$18a''$	$11a'' \rightarrow 59a'$	70		z	−0.1361	0.0185	0.0022
4.92	$16a'$	$10a'' \rightarrow 12a''$	73		x	0.0242	0.0006	0.0002
		$9a'' \rightarrow 12a''$	14		y	−0.0380	0.0014	
4.96	$17a'$	$50a' \rightarrow 54a'$	59		x	0.0269	0.0007	0.0001
		$50a' \rightarrow 56a'$	16		y	−0.0022	0.0000	
5.04	$19a''$	$10a'' \rightarrow 54a'$	20		z	0.1807	0.0326	0.0040
		$10a'' \rightarrow$ mix						

Table S6: Excited states of *op*-DHBDI⁻ continued

EE	Sym	Exc	c^2	Exc	Comp	TDM	Trans. str.	f
5.09	$20a''$	$50a' \rightarrow 13a''$	85		z	0.0086	0.0001	0.0000
5.15	$18a'$	$11a'' \rightarrow 16a''$	35		x	−0.1115	0.0124	0.0016
		$11a'' \rightarrow 19a''$	11		y	0.0226	0.0005	
5.16	$19a'$	$50a' \rightarrow 59a'$	18		x	−0.0069	0.0000	0.0019
		$50a' \rightarrow$ mix			y	−0.1226	0.0150	
5.16	$20a'$	$49a' \rightarrow 52a'$	60		x	0.1516	0.0230	0.0054
					y	0.1414	0.0200	
5.20	$21a''$	$10a'' \rightarrow 55a'$	38		z	−0.0033	0.0000	0.0000
		$10a'' \rightarrow 58a'$	11					
5.21	$22a''$	$50a' \rightarrow 19a''$	20		z	−0.0252	0.0006	0.0001
		$50a' \rightarrow 16a''$	16					
5.23	$23a''$	$11a'' \rightarrow 61a'$	27		z	0.1142	0.0130	0.0017
		$11a'' \rightarrow 60a'/63a'$	24/23					
5.29	$21a'$	$50a' \rightarrow 60a'$	27		x	0.0488	0.0024	0.0005
		$50a' \rightarrow 63a'$	18		y	0.0381	0.0015	
5.29	$24a''$	$49a' \rightarrow 12a''$	89		z	−0.0314	0.0010	0.0001
5.33	$25a''$	$11a'' \rightarrow 62a'$	29		z	−0.0771	0.0060	0.0008
		$11a'' \rightarrow 64a'$	15					
5.35	$26a''$	$9a'' \rightarrow 52a'$	30		z	−0.0816	0.0067	0.0009
5.39	$22a'$	$49a' \rightarrow 53a'$	30		x	−0.1571	0.0247	0.0094
		$49a' \rightarrow 56a'$	17		y	0.2157	0.0465	
5.41	$27a''$	$9a'' \rightarrow 53a'$	40		z	0.0283	0.0008	0.0001
5.42	$28a''$	$11a'' \rightarrow 61a'$	23		z	0.0938	0.0088	0.0012
		$11a'' \rightarrow 62a'$	20					
5.44	$23a'$	$9a'' \rightarrow 12a''$	50	$\pi_2 \rightarrow \pi^*_{\text{cont}}$	x	0.4347	0.1889	0.0273
		$8a'' \rightarrow 12a''$	14		y	−0.1257	0.0158	
5.48	$29a''$	$10a'' \rightarrow 54a'$	32		z	−0.1036	0.0107	0.0014
		$9a'' \rightarrow 52a'$	14					
5.48	$24a'$	$8a'' \rightarrow 14a''$	41	$\pi_1 \rightarrow \pi^*_1$	x	−0.4023	0.1618	0.0310
					y	0.2633	0.0693	
5.51	$30a''$	$11a'' \rightarrow 64a'$	37		z	0.0689	0.0047	0.0006
5.54	$25a'$	$11a'' \rightarrow 17a''$	37	$\pi_4 \rightarrow \pi^*_{\text{cont}}$	x	−0.5380	0.2895	0.0518
		$10a'' \rightarrow 13a''$	18	$\pi_3 \rightarrow \pi^*_{\text{cont}}$	y	0.3034	0.0920	
5.60	$31a''$	$10a'' \rightarrow 58a'$	29		z	−0.0832	0.0069	0.0009
		$10a'' \rightarrow 56a'$	22					
5.62	$26a'$	$8a'' \rightarrow 14a''$	20	$\pi_1 \rightarrow \pi^*_1$	x	0.3555	0.1264	0.0707
		$10a'' \rightarrow 16a''$	15		y	0.6222	0.3871	
5.62	$27a'$	$10a'' \rightarrow 13a''$	40	$\pi_3 \rightarrow \pi^*_{\text{cont}}$	x	−0.2177	0.0474	0.0129
		$11a'' \rightarrow 17a''$	21		y	0.2155	0.0464	
5.64	$28a'$	$50a' \rightarrow 59a'$	36		x	0.1307	0.0171	0.0025
		$50a' \rightarrow 57a'$	21		y	0.0308	0.0009	
5.70	$32a''$	$10a'' \rightarrow 58a'$	14		z	0.0188	0.0004	0.0000
5.74	$33a''$	$50a' \rightarrow 15a''$	38		z	0.0229	0.0005	0.0001
		$50a' \rightarrow 21a''/27a''$	13/11					
5.80	$34a''$	$11a'' \rightarrow 66a'$	21		z	−0.0238	0.0006	0.0001
		$11a'' \rightarrow 67a'/68a'$	12/11					
5.82	$35a''$	$10a'' \rightarrow 57a'$	27		z	−0.0008	0.0000	0.0000
		$10a'' \rightarrow 58a'$	11					

9 Optimised geometries

***p*-HBDI⁻**

C	-3.359244	-0.662209	0.000000
C	-4.298734	0.459330	0.000000
C	-1.983594	-0.497837	0.000000
C	-1.395420	0.814711	0.000000
C	-2.293956	1.936353	0.000000
C	-3.670498	1.777230	0.000000
O	-5.557137	0.297195	0.000000
C	0.000000	1.062836	0.000000
C	1.088965	0.186386	0.000000
N	1.049995	-1.220350	0.000000
C	2.316255	-1.609986	0.000000
N	3.211827	-0.550008	0.000000
C	2.475293	0.662364	0.000000
O	3.002495	1.795981	0.000000
H	-1.860130	2.945422	0.000000
H	-1.317011	-1.364819	0.000000
H	-3.796886	-1.667696	0.000000
H	-4.336878	2.647689	0.000000
C	2.774279	-3.033462	0.000000
H	3.385022	-3.263469	0.891225
H	1.885568	-3.679246	0.000000
H	3.385022	-3.263469	-0.891225
C	4.657422	-0.602526	0.000000
H	5.004328	0.441627	0.000000
H	5.042962	-1.114087	0.897864
H	5.042962	-1.114087	-0.897864
H	0.304822	2.120097	0.000000

***cis o*-HBDI⁻**

C	-3.704081	-0.506681	0.000000
C	-4.356568	0.718994	0.000000
C	-2.245696	-0.649930	0.000000
C	-1.503288	0.628114	0.000000
C	-2.226504	1.870251	0.000000
C	-3.615339	1.939406	0.000000
H	-5.454233	0.748311	0.000000
C	-0.078915	0.763382	0.000000
C	1.036682	-0.070470	0.000000
N	1.167255	-1.472601	0.000000
C	2.465692	-1.704223	0.000000
N	3.245903	-0.546900	0.000000
C	2.379143	0.562941	0.000000
O	2.765075	1.749709	0.000000
H	-1.637448	2.797099	0.000000
O	-1.701490	-1.788747	0.000000
H	-4.273894	-1.443635	0.000000
H	-4.127351	2.906918	0.000000
C	3.083355	-3.066328	0.000000
H	3.716159	-3.224493	0.891500
H	2.269446	-3.803832	0.000000
H	3.716159	-3.224493	-0.891500
C	4.687852	-0.437981	0.000000
H	4.918991	0.637712	0.000000
H	5.128058	-0.904247	0.897543
H	5.128058	-0.904247	-0.897543
H	0.255855	1.813517	0.000000

***op*-DHBDI⁻**

C	-3.381316	-0.689048	0.000000
C	-4.295001	0.440116	0.000000
C	-1.999062	-0.566811	0.000000
C	-1.373850	0.750245	0.000000
C	-2.281870	1.881278	0.000000
C	-3.651713	1.757265	0.000000
O	-5.553692	0.307150	0.000000
C	-0.000503	1.069078	0.000000
C	1.184204	0.312067	0.000000
N	1.283308	-1.084076	0.000000
C	2.577586	-1.376725	0.000000
N	3.358984	-0.236899	0.000000
C	2.513963	0.908993	0.000000
O	2.941345	2.085518	0.000000
H	-1.824451	2.878774	0.000000
O	-1.286332	-1.731565	0.000000
H	-3.811196	-1.695724	0.000000
H	-4.301878	2.638680	0.000000
C	3.151285	-2.756031	0.000000
H	3.778332	-2.935515	0.891399
H	2.321037	-3.475810	0.000000
H	3.778332	-2.935515	-0.891399
C	4.803926	-0.153302	0.000000
H	5.049252	0.919114	0.000000
H	5.234052	-0.626607	0.898218
H	5.234052	-0.626607	-0.898218
H	0.215363	2.147608	0.000000
H	-0.301901	-1.567387	0.000000

References

[1] D. B. G. Williams and M. Lawton, *J. Org. Chem.*, 2010, **75**, 8351–8354.

[2] J. S. Paige, K. Y. Wu and S. R. Jaffrey, *Science*, 2011, **333**, 642–646.

[3] K.-Y. Chen, Y.-M. Cheng, C.-H. Lai, C.-C. Hsu, M.-L. Ho, G.-H. Lee and P.-T. Chou, *J. Am. Chem. Soc.*, 2007, **129**, 4535–4535.

[4] F. Bailly, C. Maurin, E. Teissier, H. Vezin and P. Cotelle, *Bioorg. Med. Chem.*, 2004, **12**, 5611–5618.

[5] T. Chatterjee, M. Mandal, A. Das, K. Bhattacharyya, A. Datta and P. K. Mandal, *J. Phys. Chem. B*, 2016, **120**, 3503–3510.

[6] Y.-H. Hsu, Y.-A. Chen, H.-W. Tseng, Z. Zhang, J.-Y. Shen, W.-T. Chuang, T.-C. Lin, C.-S. Lee, W.-Y. Hung and B.-C. Hong, *J. Am. Chem. Soc.*, 2014, **136**, 11805–11812.
